# Chemical novelty facilitates herbivore resistance and biological invasions in some introduced plant species

**DOI:** 10.1002/ece3.6575

**Published:** 2020-07-23

**Authors:** Brian E. Sedio, John L. Devaney, Jamie Pullen, Geoffrey G. Parker, S. Joseph Wright, John D. Parker

**Affiliations:** ^1^ Department of Integrative Biology University of Texas at Austin Austin TX USA; ^2^ Smithsonian Tropical Research Institute Ancón Republic of Panama; ^3^ Center for Biodiversity and Drug Discovery Instituto de Investigaciones Científicas y Servicios de Alta Tecnología‐AIP Ancón Republic of Panama; ^4^ Smithsonian Environmental Research Center Edgewater MD USA

**Keywords:** antiherbivore defense, coexistence, forest ecology, invasive species, mass spectrometry, molecular network

## Abstract

Ecological release from herbivory due to chemical novelty is commonly predicted to facilitate biological invasions by plants, but has not been tested on a community scale. We used metabolomics based on mass spectrometry molecular networks to assess the novelty of foliar secondary chemistry of 15 invasive plant species compared to 46 native species at a site in eastern North America. Locally, invasive species were more chemically distinctive than natives. Among the 15 invasive species, the more chemically distinct were less preferred by insect herbivores and less browsed by deer. Finally, an assessment of invasion frequency in 2,505 forest plots in the Atlantic coastal plain revealed that, regionally, invasive species that were less preferred by insect herbivores, less browsed by white‐tailed deer, and chemically distinct relative to the native plant community occurred more frequently in survey plots. Our results suggest that chemically mediated release from herbivores contributes to many successful invasions.

## Graphical abstract

Ecological release from herbivory due to chemical novelty is predicted to facilitate biological invasions by plants, but has not been tested on a community scale. We used metabolomics to assess the chemical novelty of invasive plant species at a site in eastern North America. Locally, invasive species were more chemically distinctive than natives. Among the invasive species, the more chemically distinct were less preferred by insect herbivores, less browsed by deer, and occurred more frequently in a large, regional network of forest plots. Our results suggest that chemically mediated release from herbivores contributes to many successful invasions.

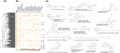

## 1 INTRODUCTION

Biological invasions occur when species are introduced to regions outside their native ranges and subsequently experience rapid increases in relative abundance. Invasive species often suppress native species, alter ecosystem function, and carry enormous economic costs (Pyšek et al., 2012). The deliberate and accidental introduction of plants and animals to exotic geographic regions on an interhemispheric scale began in earnest in the 15th century and has continued to accelerate (Pyšek et al., 2012), yet only some introduced species become invasive. Understanding why some species become invasive and identifying traits associated with invasiveness remain imperative in community ecology (Colautti, Parker, & Cadotte, 2014).

The enemy release hypothesis posits that biological invasions are driven by escape from pests and pathogens in the introduced range (Keane & Crawley, 2002). The novel weapons hypothesis attributes this release to novel secondary metabolites that deter competitors (Callaway & Aschehoug, 2000; Callaway & Ridenour, 2004) or enemies (Cappuccino & Arnason, 2006; Lind & Parker, 2010; Verhoeven, Biere, Harvey, & Van Der Putten, 2009) in the introduced range with which they do not share a coevolutionary history. Examination of the chemical novelty of invasive species thus far has been relatively limited, including the comparison of a few major compounds in many species (e.g., Cappuccino & Arnason, 2006), investigation of chemical novelty in a single species (e.g., Enge, Nylund, Harder, & Pavia, 2012), and the comparison of chemically diverse metabolomes in a few closely related species (e.g., Macel, de Vos, Jansen, van der Putten, & van Dam, 2014). The results of these studies have been consistent with the predictions of the novel weapons hypothesis, whereas a study that evaluated the effect of chemical extracts on herbivore diet preference in the absence of detailed chemical data (Lind & Parker, 2010) showed more equivocal results.

Many thousands of plant metabolites influence the interactions between a community of plants and their herbivores and pathogens, many of these compounds are likely to be shared by only a few species in an ecological community, and the structures of most remain unknown (Wang et al., 2016). This combination of vast diversity, rarity, and unknown molecular structure of plant secondary metabolites has posed a considerable challenge to understanding how chemical novelty alters herbivory across many species in a community (Burkepile & Parker, 2017; Sedio, 2017). However, recent innovations in mass spectrometry (MS) bioinformatics make it possible to compare the structures of thousands of unknown metabolites from diverse chemical classes in tens or hundreds of plant species simultaneously (Sedio,^2017^; Wang et al., 2016). Here, we take advantage of these methods to acquire and assemble tandem mass spectra (MS/MS) into molecular networks that quantify the structural similarity of all compounds across a range of plant species from numerous families and diverse chemical backgrounds, but from the same community. Moreover, by quantifying the structural similarity of all pairwise combinations of compounds, these molecular networks allow for quantification of chemical similarities between species even though few compounds are unambiguously identified (Sedio, Boya, & Rojas Echeverri, , 2018; Sedio, Echeverri, Boya, & Wright, 2017; Sedio, Parker, McMahon, & Wright, 2018).

We used MS/MS molecular network metabolomics to assess chemical similarity among 46 native and 15 invasive plant species at a mid‐Atlantic forest in Maryland to test predictions of the novel weapons hypothesis. At the local scale in Maryland, we ask: (a) Are invasive plant species less chemically similar to the native community than are native plant species? and (b) Do chemically novel or distinctive species avoid herbivory by the Japanese beetle (*Popillia japonica*) and woolly bear caterpillar (*Pyrrharctia isabella*), two common insect herbivores at the site, and by white‐tailed deer, the predominant large herbivore in the region? We then relate species variation in chemical distinctiveness at the single site in Maryland to vegetation census plots across the North American Atlantic coastal plain to ask: (c) Is variation in the frequency of invasion at a regional scale related to variation in chemical distinctiveness among the 15 invasive species?

## 2 MATERIALS AND METHODS

### 2.1 Study site and species

The Smithsonian Environmental Research Center (SERC) is located in eastern deciduous forest along the western shore of Chesapeake Bay, near Edgewater, MD (38°53′N, 76°33′W). Most forests at SERC, including those in this study, are 75‐ to 120‐year‐old mid‐successional forests and contain species broadly typical of those in the mid‐Atlantic United States (Lemoine et al., 2015). The overstory is dominated by *Liriodendron tulipifera* (tulip poplar, Magnoliaceae), *Liquidambar styraciflua* (sweetgum, Altingiaceae), *Fagus grandifolia* (American beech, Fagaceae), *Quercus* spp. (oaks, Fagaceae), *Carya* spp. (hickories, Juglandaceae), and *Fraxinus pennsylvanica* (green ash, Oleaceae). The most abundant plants in the understory are juvenile *F. grandifolia*, *Carpinus caroliniana* (American hornbeam, Betulaceae), and *Lindera benzoin* (spicebush, Lauraceae). Introduced, invasive species comprise vines, woody shrubs, and a grass common to much of the eastern United States, including *Berberis thunbergii* (Japanese barberry, Berberidaceae), *Lonicera japonica* (Japanese honeysuckle, Caprifoliaceae), *Microstegium vimineum* (Japanese stiltgrass, Poaceae), *Rosa multiflora* (multiflora rose, Rosaceae), and *Rubus phoenicolasius* (wineberry, Rosaeceae), among others (Lemoine et al., 2015).

In the present study, we included 46 native plant species that represent 70.8% of the native species and 98.5% of the native stems ≥ 1 cm diameter at breast height (dbh) recorded in a 16‐ha, mapped Forest Dynamics Plot (FDP) located at SERC, as well as 37.5% of the native species and 30.3% of the native species cover recorded in an understory census of the same plot. In addition, we sampled 15 invasive species that represent 90% of the invasive species and 99.8% of invasive stems recorded in the SERC FDP, and 91.7% of the invasive species and 98.9% of the invasive species cover recorded in the SERC understory census. All 15 introduced species are considered “invasive” by state or federal agencies (https://www.invasivespeciesinfo.gov).

### 2.2 Local abundance and regional occurrence frequency

In order to measure the local abundance of woody species, we estimated abundances based on basal area in the 2014 SERC FDP census of stems ≥ 1 cm dbh. To measure the local abundance of small‐statured and herbaceous species, we used a census of understory vegetation cover in 90 1‐m^2^ plots carried out in 2016. For both abundance measures, woody basal area and understory vegetation cover, we scaled species‐level abundances from 0 to 1, where 0 and 1 corresponded to absent species and the most abundant species in each census. In order to measure local abundances on a comparable scale for species ranging from a grass (*M. vimineum*) to overstory trees, we recorded “abundance” as whichever abundance value, scaled basal area or scaled understory cover, was greatest for each species.

In addition to local abundance at SERC, we estimated invasion on a regional scale for the 15 invasive species by recording their occurrence in the U.S. Department of Agriculture Forest Inventory and Analysis (FIA) database (Bechtold & Scott, 2005) in the Atlantic coastal plain, defined as plots occurring south of 40.2°N, east of 80°W, and below 500 m elevation in the states of New Jersey, Delaware, Maryland, Virginia, and North Carolina. We considered a total of 2,505 FIA “invasive species” subplots (see Bechtold & Scott, 2005). For each species, we recorded the proportion of the plots in which the species had ever been recorded since 2005 (Table 1).

**TABLE 1:** Status, CSCS_native_, diet preference, proportion browsed, and proportion of FIA plots occupied

Latin name	Status	CSCS_native_	Japanese beetle diet preference	Woolly bear caterpillar diet preference	Proportion browsed by white‐tailed deer	Number of individuals observed for deer browse	Proportion of FIA plots occupied
*Acer negundo*	Native	0.084	—	0.50	0.86	35	—
*Acer rubrum*	Native	0.077	0.59	0.49	0.50	12	—
*Ailanthus altissima*	Invasive	0.097	0.26	0.46	0.60	10	0.9313
*Albizia julibrissin*	Invasive	0.091	0.27	0.11	0.77	13	0.4898
*Amelanchier arborea*	Native	0.081	—	—	0.00	1	—
*Arisaema triphyllum*	Native	0.112	—	0.54	0.00	7	—
*Asimina triloba*	Native	0.119	—	0.19	0.00	25	—
*Berberis thunbergii*	Invasive	0.047	0.06	0.32	0.15	20	0.5864
*Boehmeria cylindrica*	Native	0.079	—	—	0.23	35	—
*Campsis radicans*	Native	0.118	—	—	0.71	17	—
*Carpinus caroliniana*	Native	0.074	—	0.47	0.89	19	—
*Carya alba*	Native	0.186	—	—	0.42	31	—
*Carya cordiformis*	Native	0.141	—	—	0.58	33	—
*Carya glabra*	Native	0.174	—	—	0.50	10	—
*Celastrus orbiculatus*	Invasive	0.095	0.25	0.12	0.61	28	0.8248
*Celtis occidentalis*	Native	0.035	—	—	0.86	22	—
*Cornus florida*	Native	0.155	0.37	0.20	0.51	35	—
*Diospyros virginiana*	Native	0.107	—	—	0.46	13	—
*Elaeagnus umbellata*	Invasive	0.054	0.31	0.75	0.76	17	0.0032
*Fagus grandifolia*	Native	0.143	0.64	0.65	0.40	25	—
*Fraxinus pennsylvanica*	Native	0.126	—	—	0.40	15	—
*Hedera helix*	Invasive	0.115	—	—	0.14	22	0.0052
*Ilex opaca*	Native	0.141	—	—	0.69	32	—
*Juglans nigra*	Native	0.105	—	—	0.52	25	—
*Kalmia latifolia*	Native	0.092	—	—	0.71	28	—
*Ligustrum vulgare*	Invasive	0.126	—	—	0.84	32	0.0016
*Lindera benzoin*	Native	0.088	0.12	0.28	0.73	40	—
*Liquidambar styraciflua*	Native	0.183	0.67	0.67	0.26	50	—
*Liriodendron tulipifera*	Native	0.073	0.11	0.22	0.36	25	—
*Lonicera japonica*	Invasive	0.053	0.10	0.60	0.55	47	0.9892
*Lonicera maackii*	Invasive	0.056	0.48	—	0.88	32	0.0080
*Microstegium vimineum*	Invasive	0.098	—	0.67	0.00	23	0.9844
*Nyssa sylvatica*	Native	0.188	—	—	1.00	8	—
*Parthenocissus quinquefolia*	Native	0.043	0.32	0.60	0.14	14	—
*Paulownia tomentosa*	Invasive	0.062	0.42	0.14	0.00	11	0.6762
*Persicaria perfoliata*	Invasive	0.066	0.52	0.34	0.36	36	—
*Platanus occidentalis*	Native	0.148	—	0.69	0.05	19	—
*Prunus serotina*	Native	0.105	—	—	0.76	17	—
*Pueraria montana*	Invasive	0.107	—	0.86	0.11	36	0.2287
*Quercus alba*	Native	0.144	0.62	—	0.23	13	—
*Quercus coccinea*	Native	0.182	—	—	—	0	—
*Quercus falcata*	Native	0.162	—	—	0.42	12	—
*Quercus marilandica*	Native	0.125	—	—	0.50	22	—
*Quercus michauxii*	Native	0.092	—	—	0.32	22	—
*Quercus palustris*	Native	0.164	—	—	0.50	6	—
*Quercus rubra*	Native	0.157	—	—	0.71	17	—
*Quercus velutina*	Native	0.102	—	—	0.23	44	—
*Rhus typhina*	Native	0.057	—	—	—	0	—
*Rosa multiflora*	Invasive	0.160	0.67	0.61	0.92	48	0.0295
*Rubus allegheniensis*	Native	0.137	0.42	—	0.46	46	—
*Rubus occidentalis*	Native	0.152	—	0.44	0.46	13	—
*Rubus phoenicolasius*	Invasive	0.137	0.43	0.83	0.71	41	0.0000
*Sambucus nigra*	Native	0.124	—	—	1.00	5	—
*Sassafras albidum*	Native	0.130	—	—	0.88	25	—
*Smilax rotundifolia*	Native	0.136	0.58	0.65	0.94	31	—
*Toxicodendron radicans*	Native	0.077	0.56	0.55	0.19	31	—
*Ulmus rubra*	Native	0.090	—	—	0.81	21	—
*Viburnum acerifolium*	Native	0.129	—	—	0.00	1	—
*Viburnum dentatum*	Native	0.142	—	—	1.00	3	—
*Viburnum prunifolium*	Native	0.085	—	0.01	0.89	27	—
*Vitis vulpina*	Native	0.082	—	—	0.72	18	—

Note

CSCS_native_ is the mean chemical structural–compositional similarity to the native flora at SERC, weighted by the relative abundance of native species. Proportion of FIA plots occupied is the proportion of 2,505 “invasive species” subplots occupied by that species in the US Department of Agriculture Forest Inventory and Analysis network across the Atlantic coastal plain in the states of New Jersey, Delaware, Maryland, Virginia, and North Carolina.

### 2.3 Liquid Chromatography–Tandem Mass Spectrometry (LC‐MS/MS)

We collected 231 leaf samples from the 15 invasive and 46 native species from April to August 2014 (Sedio, Boya, et al., 2018). Sample collection, chemical extraction, and analysis methods were identical to those reported by Sedio et al. (2017), Sedio, Boya, et al. (2018), Sedio, Parker, et al. (2018). Briefly, we employed a 90:10 methanol:water pH 5 solvent to extract small organic molecules of a wide range in polarity. Mild acidity aids the extraction of alkaloids. Each sample was analyzed individually using ultra‐high‐performance liquid chromatography (UHPLC), electrospray ionization and fragmentation, and tandem mass spectrometry (MS/MS; Sedio et al., 2017). We used reverse phase UHPLC (Agilent Technologies) with a flow rate of 0.5 ml/min at 25°C. We developed a 37 min solvent gradient to separate compounds characterized by a wide range of polarities. The solvent gradient included a 25 min gradient from 5% to 100% acetonitrile, followed by 8 min of isocratic 100% acetonitrile using a Kinetex C18 UHPLC column with 100 mm length, 2.1 mm internal diameter, and 1.7 μm particle size (Phenomenex). Both solvents included 0.1% formic acid to facilitate protonation. Liquid chromatographic separation was followed by mass spectrometry detection using electrospray ionization (ESI) in positive mode on a micrOTOF‐QIII quadrupole time‐of‐flight mass spectrometer (Bruker Daltonics). Data‐dependent collision energy, acquisition time, and other parameters were optimized to detect and fragment molecules over as wide a range in the mass to charge ratio (*m*/*z*) of the parent compound as possible (ca. 150 *m*/*z* to > 1,600 *m*/*z*).

### 2.4 Molecular networking and bioinformatics

MS/MS spectra of fragmented molecules were clustered into a consensus spectrum that represented a single unique molecular structure using the Global Natural Products Social (GNPS) Molecular Networking software (gnps.ucsd.edu; Wang et al., 2016). We refer to consensus spectra as compounds throughout. Molecules with similar structures fragment into many of the same substructures. Thus, the similarity of mass to charge ratio (*m*/*z*) of the fragments of two molecules reflects their structural similarity. We quantified structural similarity for every pair of compounds from all 61 species as the cosine of the angle between vectors defined by the *m*/*z* values of their constituent fragments (Wang et al., 2016). Cosine values < 0.6 are unlikely to reflect meaningful levels of chemical structural similarity and were zeroed (Wang et al., 2016). Our MS data can be found at http://gnps.ucsd.edu/ProteoSAFe/status.jsp?task=d1f7f083fa554f2c9608f238c1ccda0e (Sedio, Parker, et al., 2018).

To visualize the chemical space occupied by the MS/MS spectra and the species that possess them, we used the online molecular networking workflow at GNPS (gnps.ucsd.edu; Wang et al., 2016). This visualization approach represents each MS/MS spectrum as a node and spectrum‐to‐spectrum relatedness as edges (connections) between nodes. The data were filtered by removing all MS/MS peaks within ±17 Da of the precursor *m*/*z*. MS/MS spectra were window filtered by choosing only the top six peaks in the ±50 Da window throughout the spectrum (Watrous et al., 2012). The data were then clustered with MS‐Cluster (Frank et al., 2008) with a parent mass tolerance of 2.0 Da and a MS/MS fragment ion tolerance of 0.5 Da to create consensus spectra. Further, consensus spectra that contained only one spectrum were discarded. A network was then created where edges were filtered to have a cosine similarity score above 0.6 and more than six matched peaks (Sedio, Boya, et al., 2018; Sedio et al., 2017; Sedio, Parker, et al., 2018; Watrous et al., 2012). Further, edges between two nodes were kept in the network only if each of the nodes appeared in each other's respective top ten most similar nodes.

In addition, to GNPS molecular networking, we identified and classified compounds using the “feature‐based molecular networking” workflow (Tripathi et al., 2020). Due to limitations of memory and computation time, we restricted the “feature‐based” analyses to abundant compounds with ion intensity >20,000. We identified compounds found in the 15 invasive species by aligning LC retention time and MS spectra using MZMine 2 (Pluskal, Castillo, Villar‐Briones, & Orešič, 2010). We then matched isotopic patterns and MS/MS spectra to those of known compounds in the PubChem public spectral library (https://pubchem.ncbi.nlm.nih.gov/) using the software SIRIUS 4 (Dührkop et al., 2019). Finally, we used Qemistree (Tripathi et al., 2020) to represent the structural similarity of abundant compounds as a phylogeny‐like tree and ClassyFire (Feunang et al., 2016) to classify compounds based on chemotaxonomy.

Our methods detect both primary metabolites involved in core metabolic pathways, which tend to be conserved across most plants, and secondary metabolites involved in signaling, species interactions, and defense. Secondary metabolites will dominate the combined metabolomes of a phylogenetically diverse sample of 61 species due to their greater diversity and much greater interspecific variability (Salminen & Karonen, 2011).

### 2.5 Chemical structural and compositional similarity (CSCS)

Sedio et al. (2017) developed a metric that quantifies chemical structural–compositional similarity (CSCS) over all compounds in two samples, with every compound weighted by its relative ion intensity or concentration in each sample. CSCS accounts for the presence and concentration of structurally similar compounds that are not shared between samples. A simple example illustrates the implications. Compounds *x* and *y* are structurally similar, species *A* contains compound *x* but not *y*, and species *B* contains *y* but not *x*. In this example, compounds *x* and *y* make no contribution to a conventional method of calculating similarity, such as Bray–Curtis similarity, but make a positive contribution to CSCS proportional to their structural similarity and concentrations.

We calculated the species mean ion intensity for every compound over conspecific individuals. We then standardized species‐level chemical composition by species‐specific total ion intensities and calculated CSCS for every pair of species using all compounds in the dataset, including those that remained unlinked to any other compound in the network. Given 61 species, there are $\left(\begin{array}{c} 61\\ 2 \end{array}\right)=3,660$ pairs of species. For each invasive species, we then calculated the mean value of CSCS relative to all native species, weighted by abundance (Table 1). For native species, we calculated the abundance‐weighted mean value of CSCS relative to all other native species (Table 1). The abundance‐weighted metric was strongly correlated with the unweighted metric (correlation 0.98, *R* ^2^ = .96). We refer to each species’ abundance‐weighted mean CSCS relative to native species at SERC as CSCS_native_ throughout.

### 2.6 Insect herbivore diet assays

To examine how plant chemical similarity affected feeding preference of insect herbivores, we assessed the diet preferences of two generalist insect herbivores that are locally common at SERC: the Japanese beetle, *P. japonica,* and the woolly bear caterpillar, *P. isabella*. *Popillia japonica* is a major pest species, is considered invasive in North America, and maintains a broad diet of 300 plant species from 79 families (Fleming, 1972). *Pyrrharctia isabella* is a native generalist herbivore in eastern deciduous forests of North American and feeds on a wide variety of plants including many with deterrent secondary chemistry (Wagner, 2005).

Woolly bear feeding trial results were reported by Lind and Parker (2010). We employed an identical method to assay the feeding preferences of the Japanese beetle. Adult *P. japonica* were field collected at SERC in July 2016 using standard traps (Spectracide Bag‐a‐bug Japanese beetle traps, United Industries Corporation) that were placed on various host plants in forest and edge habitats. While in captivity, individuals were held at room temperature and fed fresh *Platanus occidentalis* leaves daily. Beetles were used in feeding assays within 2 days of collection, and no individual was used more than once.

For feeding assays, we incorporated total crude leaf chemistry extracts into artificial diets. We extracted leaf chemistry from 25 plant species that are abundant at SERC, and in surrounding areas (Parker, Burkepile, Lajeunesse, & Lind, 2011), 13 species native to eastern Maryland and 12 invasive species (Table 1). Leaf samples used in Japanese beetle assays were the same samples analyzed using LC‐MS/MS. To prepare plant chemical extracts, we followed methods outlined in Lind and Parker (2010) and Lemoine, Drews, Burkepile, and Parker (2013). Frozen leaf tissues were coarsely ground, added to a beaker, and extracted with a series of hydrophilic to lipophilic solvents (1:1 v/v of water:methanol, 2:1 v/v of methanol:dichloromethane, and 2:1 v/v dichloromethane:methanol). Extraction times were 2, 2, and 12 hr for each step, respectively. The three extracts were combined, condensed using a vacuum centrifuge, resuspended in 5 ml acetone, and added to an artificial diet mixture of 1 g wheat germ, 1 g cellulose, and 0.025 g FABCO‐1 antifungal agent. Extracts from 2 g of dried leaves were added to 2 g of artificial diet preserving the natural ratio of secondary metabolites to food mass. Twenty ml of boiling water was added to the diet and the mixture stirred until all acetone had evaporated. Agar powder (0.75 g) was added to the mixture to act as a solidifying agent and the mixture immediately poured into a 1.5 cm wide mold. Control foods lacking chemical extracts were prepared in an identical fashion using acetone without chemical extracts.

For each plant species feeding assay (*n* = 20 assays per plant species), 1 cm^2^ strips of control and chemically treated agar foods were weighed to the nearest milligram and placed on opposite sides of a petri dish. A single, adult *P. japonica* individual was then placed in the center of each petri dish, and the lid was closed. Each feeding trial had a control petri dish containing test and control strips of food but no herbivores to control for mass loss that was unrelated to herbivory. Petri dishes were placed into a rearing chamber set to a constant temperature of 25°C with a 16 hr:8 hr light:dark cycle. After 24 hr, the agar strips were reweighed and diet preference was quantified as mass eaten corrected for expected mass loss due to evaporation over the course of each individual feeding trial.

### 2.7 White‐tailed deer browsing

Understory plants in the SERC forest were surveyed for deer browsing damage in September 2017. During the census, the presence or absence of deer browsing was recorded on 1,429 individual plants (<2 m height) from 15 invasive and 46 native species in the FDP plot and surrounding areas (Table 1). Each plant was separated from others of the same species by at least 10 m. We calculated the proportion of individuals browsed for each species (Table 1).

### 2.8 Plant functional traits

Physical defenses and leaf nitrogen and phosphorus levels are also important determinants of resistance to herbivory and abundance (Lemoine et al., 2015; Lind & Parker, 2010). We thus compiled % water, specific leaf area (SLA), leaf toughness (Newtons), trichome density (cm^−2^), % C, % N, and % P for 48 species (Table 2) from Lind and Parker (2010) and from new data collected using the same methods.

**TABLE 2:** Functional Traits. Data reported by Lind and Parker (2010) were supplemented with measurements on additional species

Latin Name	% Water	SLA (cm^2^/g)	Leaf Toughness (*N*)	Trichomes (cm^−2^)	% C	% N	% P
*Acer negundo*	77.4	376.6	1.01	9.15	44.67	2.56	0.24
*Acer rubrum*	59.42	261.3	1.36	0.7	48.3	1.88	0.15
*Ailanthus altissima*	76.92	391.6	1.16	57.15	45.07	3.93	0.32
*Albizia julibrissin*	63.68	209.7	1.28	80.25	47.5	3.79	0.23
*Amelanchier arborea*	53.21	397.7	1.92	115	45.91	1.653	0.224
*Arisaema triphyllum*	85.46	514.2	1.41	0.1	44.02	2.84	0.21
*Asimina triloba*	74.57	5,256	1.943	83.07	45.26	3.03	0.331
*Berberis thunbergii*	68.87	222.2	1.7	0.65	44.56	1.56	0.53
*Boehmeria cylindrica*	NA	NA	NA	NA	NA	NA	NA
*Campsis radicans*	NA	NA	NA	NA	NA	NA	NA
*Carpinus caroliniana*	56.43	318.2	1.15	34.35	47.51	2.18	0.18
*Carya alba*	59.02	348.6	2.986	181.5	44.18	1.91	0.147
*Carya cordiformis*	56.10	376.3	2.745	334	45.01	2.127	0.172
*Carya glabra*	59.27	382.1	2.976	121.9	43.68	2.107	0.158
*Celastrus orbiculatus*	80.44	300.5	0.79	0.2	43.28	2.94	0.25
*Celtis occidentalis*	NA	NA	NA	NA	NA	NA	NA
*Cornus florida*	64.56	380.0	1.65	433.9	44.59	1.52	0.13
*Diospyros virginiana*	NA	NA	NA	NA	NA	NA	NA
*Elaeagnus umbellata*	70.35	253.7	1.08	465.4	48.10	3.59	0.16
*Fagus grandifolia*	50.77	346.8	1.46	118.8	47.66	2.17	0.13
*Fraxinus pennsylvanica*	68.55	452.4	2.256	106.5	44.90	2.267	0.242
*Hedera helix*	NA	NA	NA	NA	NA	NA	NA
*Ilex opaca*	59.36	104.3	8.79	6.32	47.73	1.487	0.083
*Juglans nigra*	NA	NA	NA	NA	NA	NA	NA
*Kalmia latifolia*	63.45	134.1	4.7	220	NA	NA	NA
*Ligustrum vulgare*	72.82	318.1	2.9	1.7	44.71	1.99	0.287
*Lindera benzoin*	75.52	403.22	1.47	13.7	47.89	3.28	0.27
*Liquidambar styraciflua*	72.94	300.93	1.58	1.65	47.24	1.73	0.26
*Liriodendron tulipifera*	77.72	382.92	1.55	15.8	46.87	2.2	0.15
*Lonicera japonica*	74.66	374.94	1.12	15	42.73	1.75	0.34
*Lonicera maackii*	NA	NA	NA	NA	NA	NA	NA
*Microstegium vimineum*	56.56	722.3	1.17	112.2	45.02	2.97	0.32
*Nyssa sylvatica*	74.61	467.5	1.958	5.808	44.67	2.04	0.230
*Parthenocissus quinquefolia*	79.96	416.21	1.77	12.8	44.12	2.15	0.21
*Paulownia tomentosa*	74.32	197.26	1.13	456.05	47.79	2.04	0.2
*Persicaria perfoliata*	85.65	906.32	0.81	1.3	43.94	3.39	0.29
*Platanus occidentalis*	65.2	246.6	1.26	0	48.98	2.53	0.21
*Prunus serotina*	66.76	294.6	3.574	4.48	45.19	2.067	0.196
*Pueraria montana*	68.5	320.3	1.62	895.4	46.66	4.85	0.31
*Quercus alba*	40.04	291.2	2.728	669.1	46.58	2.187	0.185
*Quercus coccinea*	NA	NA	NA	NA	NA	NA	NA
*Quercus falcata*	50.04	217.2	2.525	295	47.47	2.257	0.151
*Quercus marilandica*	53.71	241.1	2.527	150.9	45.9	2.073	0.131
*Quercus michauxii*	59.48	327.0	2.4	270	NA	NA	NA
*Quercus palustris*	NA	NA	NA	NA	NA	NA	NA
*Quercus rubra*	57.34	284.0	1.782	15.94	46.58	2.363	0.242
*Quercus velutina*	55.93	267.9	2.043	221.1	46.89	2.077	0.135
*Rhus typhina*	NA	NA	NA	NA	NA	NA	NA
*Rosa multiflora*	62.73	417.1	1	133.6	44.04	2.08	0.17
*Rubus allegheniensis*	NA	NA	NA	NA	NA	NA	NA
*Rubus occidentalis*	65.66	228.3	0.66	290.6	46.56	2.32	0.2
*Rubus phoenicolasius*	69.75	443.2	1.3	29,651	45.89	2.74	0.22
*Sambucus nigra*	NA	NA	NA	NA	NA	NA	NA
*Sassafras albidum*	67.85	537.5	1.725	2.188	46.71	2.287	0.158
*Smilax rotundifolia*	66.48	231.4	2.01	0	47.29	1.81	0.11
*Toxicodendron radicans*	71.61	427.6	1.15	135.7	43.15	2.32	0.35
*Ulmus rubra*	63.39	380.0	1.812	93.88	40.95	1.743	0.270
*Viburnum acerifolium*	69.47	451.3	2.171	437.1	44.7	1.713	0.158
*Viburnum dentatum*	72.10	417.7	2.848	46.48	44.32	1.75	0.149
*Viburnum prunifolium*	72.93	268.75	1.06	0	43.55	1.7	0.2
*Vitis vulpina*	NA	NA	NA	NA	NA	NA	NA

Note

Traits % water, SLA, leaf toughness, and trichomes are means for 20 individuals per species. Traits %C, %N, and %P are means for five individuals per species.

### 2.9 Statistical analyses

We examined the relationship between invasive status and CSCS_native_ and between invasive status and insect diet preference and white‐tailed deer browse using ANOVA. In addition, we examined the relationship between status, CSCS_native_, and seven functional traits for 48 species using MANOVA.

Phylogenetic methods are necessary for the study of species‐level data because species are nonindependent for the purposes of statistical analyses due to their shared evolutionary history (Felsenstein, 1985; Harvey & Pagel, 1991). Phylogenetic regression assumes that the residual error in the regression model, but not the traits themselves, is distributed according to a multivariate normal distribution with variances and covariances proportional to the phylogenetic relationships among species (Revell, 2010). Hence, we employed generalized least squares regression methods and examined the phylogenetic signal in the residual error in each model based on the ForestGEO‐CTFS mega‐phylogeny (Erickson et al., 2014) using the R package “picante” (Kembel et al., 2008) in R version 3.2.3 (R Development Core Team, 2020). For models in which the residual errors for exhibited phylogenetic signal, we employed phylogenetic ANOVA or MANOVA using the R package “geiger” (Harmon, Weir, & Brock, 2008). For all other regression models, we report the *K* statistic and *p*‐value associated with the Blomberg, Garland, & Ives, (2003) test of phylogenetic signal in the residual error (Table 3).

**TABLE 3:** Beta regression statistics and Blomberg's K and test for phylogenetic signal in the residual error of each linear regression

Model	Herbivore	Beta regression R^2^	Beta regression *p*	Blomberg's *K*	Phylogenetic signal *p*
Diet preference ~ CSCS_native_ (all plants)	Japanese Beetle	**.326**	**<.001**	0.319	.792
	Woolly Bear Caterpillar	.065	.096	0.490	.370
	Insects Combined	**.225**	**.014**	0.345	.420
	White‐tailed Deer	.000	<.001	**0.172**	**.012**
Diet preference ~ CSCS_native_ (invasive species only)	Japanese Beetle	**.305**	**.001**	1.183	.927
	Woolly Bear Caterpillar	.122	.245	0.477	.879
	Insects Combined	.247	.113	0.217	.887
	White‐tailed Deer	**.106**	**.001**	0.518	.415
Proportion FIA plots occupied ~ Diet Preference	Japanese Beetle	**.463**	**.016**	0.304	.665
	Woolly Bear Caterpillar	.201	.105	**1.136**	**.013**
	Insects Combined	**.325**	**.022**	0.372	.589
	White‐tailed Deer	**.159**	**<.001**	0.231	.862
Proportion FIA plots occupied ~ CSCS_native_		**.307**	**<.001**	0.229	.870

Note

Beta regression models are considered valid if no phylogenetic signal is detected in the residual error.

Insect herbivore diet preference, the proportion of individuals browsed at SERC, CSCS_native_, and the proportion of FIA plots occupied all comprise variables represented as a proportion, and hence, we used linear regression based on the beta distribution to evaluate the relationships between these variables, using the R package “betareg” (Cribari‐Neto & Zeileis, 2010). We considered the relationship between insect diet preference and CSCS_native_ separately for Japanese beetle and woolly bear caterpillar and in a combined model including terms for CSCS_native_, insect species, and an interaction term. Regressions that included proportion browsed were weighted by the number of observations for each species.

Phylogenetic regressions and tests of phylogenetic signal were based on the ForestGEO‐CTFS mega‐phylogeny (Erickson et al., 2014), from which we pruned 57 of our 61 study species. We added two invasive species [*Persicaria perfoliata* (mile‐a‐minute weed, Polygonaceae) and *Pueraria montana* (kudzu, Fabaceae)] and two native species [*Rhus typhina* (staghorn sumac, Anacardiaceae) and *Vitis vulpina* (fox grape, Vitaceae)] to the pruned phylogeny by referencing the angiosperm phylogeny R20120829 using Phylomatic (Webb & Donoghue, 2005).

## 3 RESULTS

**FIGURE 1:**
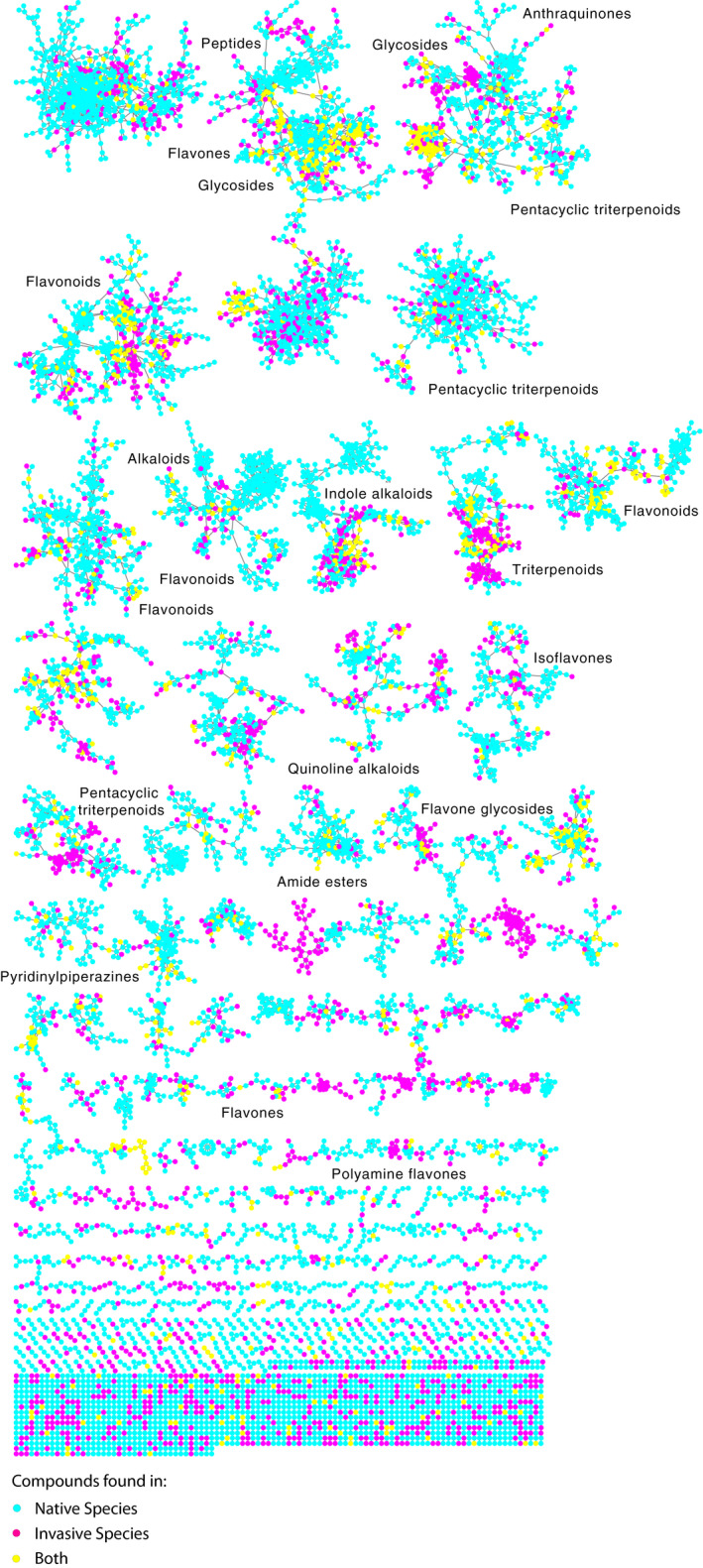
Molecular networks indicating the incidence of small molecules in invasive species only (purple), native species only (blue), and both invasive and native species (yellow) at SERC, Maryland. Nodes represent compounds; links between nodes indicate molecular structural similarity between compounds. The 85 compounds that matched a known compound in the GNPS public MS library (gnps.ucsd.edu) were used to identify the chemical class of some subnetworks (e.g., “flavonoids”). The 12,718 compounds linked to at least one other compound by a cosine similarity score ≥ 0.6 are included

**FIGURE 2:**
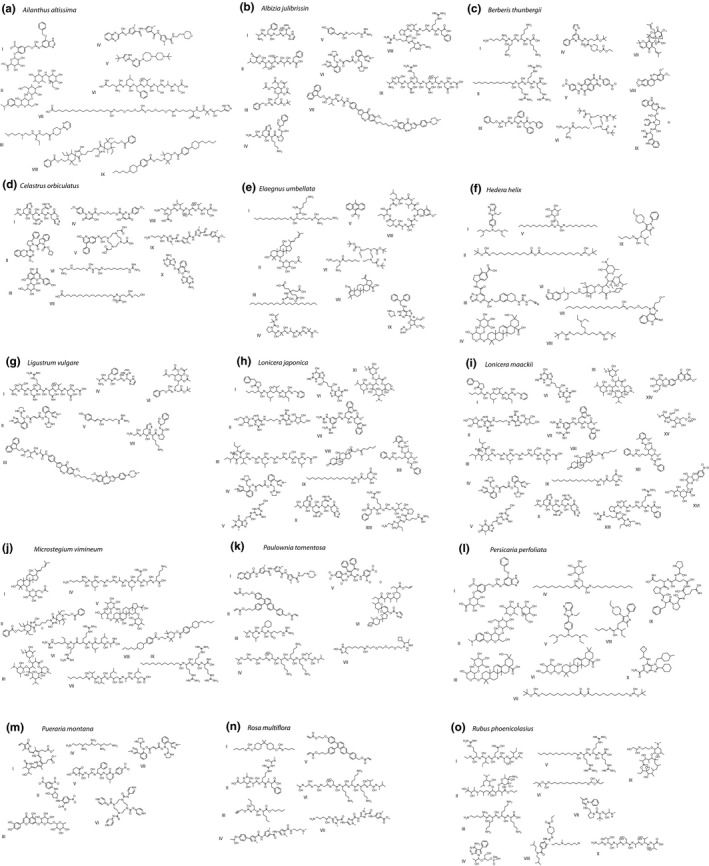
The most abundant compounds unique to each invasive species. Compounds included occur in at least one of the invasive species, but not in native species at SERC, Maryland. Each structure shown represents the best match to the PubChem database, based on isotopic patterns, molecular formulae, and MS/MS fragmentation patterns (Dührkop et al., 2019). Compounds shown here exclude simple fatty acids and chlorophyll degradation products. See Table 4 for systematic compound names

LC‐MS/MS detected 36,561 structurally unique compounds (Figure 1). 23,843 of the compounds were structurally dissimilar from all other compounds. The remaining 12,718 compounds formed 872 structurally similar networks that ranged in size from 2 to 8,758 compounds. For clarity of visualization only, we broke the 8,758‐compound network into 287 smaller networks using Markov Chain Clustering (Jäger, 2015). Thus, Figure 1 presents 1,159 networks of structurally similar compounds. Compound identifications made by matching isotopic and MS/MS fragmentation patterns included flavonoids, glycosides, piperazines, quinoline alkaloids, indole alkaloids, and terpenoids, classes known to include antiherbivore defenses (Figures 1,2; Table 4).

**TABLE 4:** Systematic compound names for the most abundant compounds unique to each of 15 invasive species

Species	Compound Name
*Ailanthus altissima*	I. 6‐(4‐((((7‐(benzyloxy)*H*‐1‐benzo[*d*]imidazol‐5‐yl)imino)(hydroxy)methoxy)methyl)‐2‐nitrophenoxy)‐3,4,5‐trihydroxytetrahydro‐2*H*‐pyran‐2‐carboxylic acid
	II. 6‐((6‐((6‐((6‐(3‐(dimethylamino)phenoxy)‐4,5‐dihydroxy‐2‐(hydroxymethyl)tetrahydro‐2*H*‐pyran‐3‐yl)oxy)‐4,5‐dihydroxy‐2‐(hydroxymethyl)tetrahydro‐2*H*‐pyran‐3‐yl)oxy)‐4,5‐dihydroxy‐2‐methyltetrahydro‐2*H*‐pyran‐3‐yl)amino)‐4‐(hydroxymethyl)cyclohex‐4‐ene‐1,2,3‐triol
	III. 2‐ethyl‐1‐(2‐((3‐methoxypropyl)(methyl)amino)ethyl)‐3‐(3‐oxo‐3‐(4‐(pyrimidin‐2‐yl)piperazin‐1‐yl)propyl)guanidine
	IV*. N*‐(1‐methyl‐5‐((1‐methyl‐5‐((1‐methyl‐5‐((2‐morpholinoethyl)carbamoyl)‐1*H*‐pyrrol‐3‐yl)carbamoyl)‐1*H*‐pyrrol‐3‐yl)carbamoyl)‐1*H*‐pyrrol‐3‐yl)quinoxaline‐2‐carbimidic acid
	V. 1‐(6‐(4‐( *tert*‐butyl)‐1*H*‐imidazol‐2‐yl)pyridin‐2‐yl)‐4‐(1‐(*tert*‐butyl)piperidin‐4‐yl)‐1,4‐diazepane
	VI. 20‐amino‐14‐benzyl‐4,7,10,13,16,19,23‐heptahydroxy‐11‐(1‐hydroxyethyl)‐2,8‐bis(hydroxymethyl)‐23‐imino‐17‐isobutyl‐5‐methyl‐3,6,9,12,15,18‐hexaazatricosa‐3,6,9,12,15,18‐hexaenoic acid
	VII. 8,17,26‐trihydroxy‐2‐((3‐((1‐hydroxy‐3‐(1*H*‐imidazol‐5‐yl)propylidene)amino)‐3‐methyl‐2‐oxobutyl)amino)‐10,13,19,22‐tetraoxa‐7,16,25‐triazatritetraconta‐7,16,25‐trienedioic acid
	VIII. (butane‐1,4‐diylbis(7,9‐diethyl‐2‐hydroxy‐6,7,9‐trimethyl‐4‐oxo‐1,3,8‐triazaspiro[4.5]dec‐1‐ene‐3,8‐diyl))bis(ethane‐2,1‐diyl) dibenzoate
	IX. 2,2,6,6‐tetramethyl‐1‐(2‐((4‐(4‐pentylcyclohexyl)benzoyl)oxy)ethyl)piperidin‐4‐yl 4‐(4‐pentylcyclohexyl)benzoate
*Albizia julibrissin*	I. 2‐((2‐amino‐1‐hydroxypropylidene)amino)‐*N*‐(2‐((di(1*H*‐imidazol‐2‐yl)methyl)imino)‐2‐hydroxyethyl)‐3‐phenylpropanimidic acid
	II. 2‐benzyl‐4,6,9,12‐tetrahydroxy‐8,11‐bis(1‐hydroxyethyl)‐5,16‐dimethyl‐14‐(oxirane‐2‐carbonyl)‐3,7,10,13‐tetraazaheptadeca‐3,6,9,12‐tetraenoic acid
	III. 2‐(acetoxymethyl)‐6‐(2‐(2‐((benzyloxy)(hydroxy)methylene)‐1‐(2‐(*tert*‐butoxy)‐2‐oxoethyl)hydrazineyl)ethyl)tetrahydro‐2*H*‐pyran‐3,4,5‐triyl triacetate
	IV. 1‐(6‐amino‐2‐((2‐((2‐amino‐1‐hydroxyethylidene)amino)‐1‐hydroxy‐3‐(1*H*‐imidazol‐5‐yl)propylidene)amino)hexanoyl)‐*N*‐(1‐oxo‐3‐phenylpropan‐2‐yl)pyrrolidine‐2‐carbimidic acid
	V*. N*‐(4‐guanidinobutyl)‐3‐(4‐hydroxyphenyl)acrylimidic acid
	VI*. O*,*O*′‐(but‐2‐enedioyl)bis(*N*‐((4,5‐dihydro‐1*H*‐imidazol‐2‐yl)methyl)‐*N*‐(2‐(1‐methyl‐1*H*‐pyrazol‐4‐yl)phenyl)hydroxylamine)
	VII. 2‐((((9*H*‐fluoren‐9‐yl)methoxy)(hydroxy)methylene)amino)‐*N*‐(1‐((4‐(8‐methoxy‐7‐((5‐((7‐methoxy‐2‐(4‐(4‐methylpiperazin‐1‐yl)phenyl)‐5‐oxo‐5,11a‐dihydro‐1*H*‐benzo[*e*]pyrrolo[1,2‐*a*][1,4]diazepin‐8‐yl)oxy)pentyl)oxy)‐10‐oxo‐3,3a,10,10a‐tetrahydrobenzo[*b*]cyclopenta[*e*]azepin‐2‐yl)phenyl)amino)‐1‐oxopropan‐2‐yl)‐3‐methylbutanimidic acid
	VIII. 1‐(1‐(2‐((2‐((2‐amino‐1‐hydroxyethylidene)amino)‐1‐hydroxy‐3‐methylpentylidene)amino)‐5‐guanidinopentanoyl)pyrrolidin‐2‐yl)‐12‐benzyl‐9‐(3‐guanidinopropyl)‐1,4,7,10‐tetrahydroxy‐3‐isopropyl‐2,5,8,11‐tetraazatrideca‐1,4,7,10‐tetraen‐13‐oic acid
	IX. 23‐amino‐5‐(carboxymethyl)‐14‐(3‐guanidinopropyl)‐4,7,10,13,16,19,22‐heptahydroxy‐11,17‐bis(2‐hydroxy‐2‐iminoethyl)‐8,20‐bis(1‐hydroxyethyl)‐3,6,9,12,15,18,21‐heptaazatetracosa‐3,6,9,12,15,18,21‐heptaenoic acid
*Berberis thunbergii*	I. 6‐amino‐2‐((6‐amino‐2‐((6‐amino‐2‐((2,6‐diamino‐1‐hydroxyhexylidene)amino)‐1‐hydroxyhexylidene)amino)‐1‐hydroxyhexylidene)amino)hexanoic acid
	II. *N*‐(1,17‐diamino‐9‐(3‐guanidinopropyl)‐8,11‐dihydroxy‐6‐(hydroxy(imino)methyl)‐1,17‐diimino‐2,7,10,16‐tetraazaheptadeca‐7,10‐dien‐12‐yl)tetradecanimidic acid III. 2‐((2‐(((benzyloxy)(hydroxy)methylene)amino)‐1‐hydroxypropylidene)amino)‐*N*‐(1‐(benzyloxy)‐1‐oxo‐3‐phenylpropan‐2‐yl)‐3‐phenylpropanimidic acid
	IV*. N*‐(5‐(*tert*‐butoxy)‐1‐(4‐((ethoxycarbonyl)oxy)piperazin‐1‐yl)‐1,5‐dioxopentan‐2‐yl)‐6‐phenyl‐4‐(1*H*‐pyrazol‐1‐yl)picolinimidic acid
	V. 2,7‐bis((4‐nitrophenyl)amino)benzo[*lmn*][3,8]phenanthroline‐1,3,6,8(2*H*,7*H*)‐tetraone
	VI. tri‐*tert*‐butyl 2,2′,2″‐(10‐(6‐(bis(2‐aminoethyl)amino)‐6‐oxohexyl)‐1,4,7,10‐tetraazacyclododecane‐1,4,7‐triyl)triacetate
	VII. 3a‐((2,2‐dimethyl‐6‐(3‐methylbut‐2‐en‐1‐yl)‐5,8‐dioxo‐5,8‐dihydro‐2*H*‐chromen‐7‐yl)oxy)‐6‐methoxy‐2,2‐dimethyl‐7a‐(3‐methylbut‐2‐en‐1‐yl)‐7‐oxo‐2,3,3a,6,7,7a‐hexahydro‐3,6‐methanobenzofuran‐4‐carboxylic acid
	VIII. 1,2‐dimethoxy‐5,6‐dihydro‐[1,3]dioxolo[4′,5′:4,5]benzo[1,2‐*c*]phenanthridine
	IX. *N* ^2^,*N* ^5^‐bis(1‐hydroxy‐1‐imino‐3‐(1*H*‐indol‐3‐yl)propan‐2‐yl)‐1‐(pyridine‐4‐yl)pyrrolidine‐2,5‐bis(carbimidic) acid
*Celastrus orbiculatus*	I. *N*‐(1‐(1*H*‐imidazol‐5‐yl)‐3‐oxobutan‐2‐yl)‐2‐((1‐hydroxy‐2‐((1‐hydroxy‐2‐((1‐hydroxyethylidene)amino)‐3‐(1*H*‐imidazol‐5‐yl)propylidene)amino)‐3‐(1*H*‐imidazol‐5‐yl)propylidene)amino)‐3‐(1*H*‐imidazol‐5‐yl)propanimidic acid
	II. methyl 2‐(1‐(1‐(cyclobutoxycarbonyl)indoline‐2‐carbonyl)‐5‐phenylpyrrolidine‐2‐carbonyl)‐1,2,3,4‐tetrahydroisoquinoline‐3‐carboxylate
	III. 5,7‐dihydroxy‐2‐(4‐hydroxyphenyl)‐8‐(3,4,5‐trihydroxy‐6‐(hydroxymethyl)tetrahydro‐2*H*‐pyran‐2‐yl)‐4*H*‐chromen‐4‐one
	IV. oxybis(ethane‐2,1‐diyl) bis(4‐hydroxy‐1‐(4‐methoxyphenyl)‐6‐oxo‐1,6‐dihydropyridazine‐3‐carboxylate)
	V. 2,2′,2″‐(10‐(2‐hydroxy‐2‐((2‐hydroxy‐4‐phenylquinolin‐7‐yl)imino)ethyl)‐1,4,7,10‐tetraazacyclododecane‐1,4,7‐triyl)triacetic acid
	VI. 2‐((((4‐((3‐aminobutyl)amino)butyl)imino)(hydroxy)methyl)amino)‐*N*‐(8‐guanidinooctyl)acetimidic acid
	VII. 18‐((1‐carboxy‐4‐hydroxy‐4‐((2‐hydroxyethyl)imino)butyl)imino)‐18‐hydroxyoctadecanoic acid
	VIII. 14‐((2‐amino‐1‐hydroxyethylidene)amino)‐11‐(*sec*‐butyl)‐4,7,10,13‐tetrahydroxy‐5,8‐bis(1‐hydroxyethyl)‐2‐(hydroxymethyl)‐3,6,9,12‐tetraazaheptadeca‐3,6,9,12‐tetraenedioic acid
	IX. 4‐amino‐*N*‐(2‐((2‐((2‐((5‐(methoxycarbonyl)‐1‐methyl‐1*H*‐pyrrol‐3‐yl)carbamoyl)‐1‐methyl‐1*H*‐imidazol‐4‐yl)carbamoyl)‐1‐methyl‐1*H*‐imidazol‐4‐yl)carbamoyl)‐1‐methyl‐1*H*‐imidazol‐4‐yl)butanimidic acid
	X. bis(6‐amino‐9*H*‐purin‐9‐yl) phthalate
*Elaegnus umbellata*	I. *N*,*N*′‐(6‐(dodecylimino)‐6‐hydroxyhexane‐1,5‐diyl)bis(2,6‐diaminohexanimidic acid)
	II. (6‐((3,12‐dihydroxy‐4,4,8,10,14‐pentamethyl‐17‐(6‐methylhepta‐2,5‐dien‐2‐yl)hexadecahydro‐1*H*‐cyclopenta[*a*]phenanthren‐6‐yl)oxy)‐3,4,5‐trihydroxytetrahydro‐2*H*‐pyran‐2‐yl)methyl acetate
	III. 4‐(2‐carboxyethyl)‐4‐((hydroxy(tricosan‐12‐ylimino)methyl)amino)heptanedioic acid
	IV. 1‐(2‐((1‐hydroxyethylidene)amino)‐2‐methylpropanoyl)‐*N*‐(6,9,12‐trihydroxy‐4,4,7,7,10,13‐hexamethyl‐3‐oxo‐2‐oxa‐5,8,11‐triazatetradeca‐5,8,11‐trien‐13‐yl)pyrrolidine‐2‐carbimidic acid
	V. methyl 10‐methylanthracene‐9‐carboxylate
	VI. tri‐*tert*‐butyl 2,2′,2″‐(10‐(6‐(bis(2‐aminoethyl)amino)‐6‐oxohexyl)‐1,4,7,10‐tetraazacyclododecane‐1,4,7‐triyl)triacetate
	VII. 10‐formyl‐5a,5b,8,8,10a‐pentamethyl‐1‐(prop‐1‐en‐2‐yl)‐2,3,4,5,5a,5b,6,7,7a,8,10a,10b,11,12,12a,12b‐hexadecahydrodicyclopenta[*a*,*i*]phenanthrene‐3a(1*H*)‐carboxylic acid
	VIII. 2‐(*sec*‐butyl)‐28‐ethyl‐3,9,18,23,26‐pentahydroxy‐8‐isobutyl‐14‐isopropyl‐17‐(4‐methoxy‐2‐methylbenzyl)‐7,13,16,20,22,22,25,29‐octamethyl‐1‐oxa‐4,7,10,13,16,19,24,27‐octaazacyclotriaconta‐3,9,18,23,26‐pentaene‐6,12,15,21,30‐pentaone
	IX. 2‐((1*H*‐1,2,4‐triazol‐1‐yl)methyl)‐5‐(6‐((2,2‐diphenylethyl)amino)‐2‐(pyrrolidin‐3‐ylimino)‐1,2‐dihydro‐9*H*‐purin‐9‐yl)tetrahydrofuran‐3,4‐diyl diformate
*Hedera helix*	I*. N* ^1^‐(2‐(diethylamino)ethyl)‐*N* ^2^,*N* ^2^‐diethyl‐*N* ^1^‐(4‐(1‐ethyl‐1*H*‐imidazo[4,5‐*b*]pyridin‐2‐yl)phenyl)ethane‐1,2‐diamine
	II. 11‐((*tert*‐butoxy(hydroxy)methylene)amino)undecanoic anhydride
	III. 1‐(5‐((((7‐(3‐(cyanomethyl)guanidino)‐5,6,7,8‐tetrahydronaphthalen‐2‐yl)methyl)imino)(hydroxy)methyl)‐[1,2,4]triazolo[1,5‐*a*]pyrimidine‐7‐carboxamido)‐4‐methyl‐2,3‐dihydro‐1*H*‐indene‐5‐carboxylic acid
	IV. 10‐((4,5‐dihydroxy‐3‐((3,4,5‐trihydroxy‐6‐methyltetrahydro‐2*H*‐pyran‐2‐yl)oxy)tetrahydro‐2*H*‐pyran‐2‐yl)oxy)‐2‐(hydroxymethyl)‐2,6a,6b,9,9,12a‐hexamethyl‐1,3,4,5,6,6a,6b,7,8,8a,9,10,11,12,12a,12b,13,14b‐octadecahydropicene‐4a(2*H*)‐carboxylic acid
	V*. N*‐(1‐hydroxy‐3‐((3,4,5‐trihydroxy‐6‐methyltetrahydro‐2*H*‐pyran‐2‐yl)oxy)‐1‐(undecylimino)propan‐2‐yl)dodecanimidic acid
	VI. 9‐((5‐(((2‐((1‐(1*H*‐indol‐6‐yl)ethyl)(ethyl)amino)ethyl)amino)methyl)‐5‐hydroxy‐4‐methoxy‐4,6‐dimethyltetrahydro‐2*H*‐pyran‐2‐yl)oxy)‐7‐((4‐(dimethylamino)‐3‐hydroxy‐6‐methyltetrahydro‐2*H*‐pyran‐2‐yl)oxy)‐3‐hydroxy‐6‐methoxy‐2,4,6,8,10‐pentamethyl‐12‐oxa‐1‐azabicyclo[11.1.1]pentadecan‐11‐one
	VII*. N*‐(2‐(2‐(4‐imino‐2‐(2‐methoxyethyl)‐4,5‐dihydro‐1*H*‐imidazo[4,5‐*c*]quinolin‐1‐yl)ethoxy)ethyl)palmitimidic acid
	VIII. di‐*tert*‐butyl (((2‐(diethylamino)ethyl)azanediyl)bis(propane‐3,1‐diyl))bis(hydrogen carbonimidate)
	IX*. N*‐(*sec*‐butyl)‐*N*′‐butyl‐*N*‐((5‐(4‐ethylpiperazin‐1‐yl)‐3‐methyl‐1‐phenyl‐1*H*‐pyrazol‐4‐yl)methyl)carbamimidic acid
*Ligustrum vulgare*	I. 23‐amino‐5‐(carboxymethyl)‐14‐(3‐guanidinopropyl)‐4,7,10,13,16,19,22‐heptahydroxy‐11,17‐bis(2‐hydroxy‐2‐iminoethyl)‐8,20‐bis(1‐hydroxyethyl)‐3,6,9,12,15,18,21‐heptaazatetracosa‐3,6,9,12,15,18,21‐heptaenoic acid
	II. *O*,*O*′‐(but‐2‐enedioyl)bis(*N*‐((4,5‐dihydro‐1*H*‐imidazol‐2‐yl)methyl)‐*N*‐(2‐(1‐methyl‐1*H*‐pyrazol‐4‐yl)phenyl)hydroxylamine)
	III. 2‐((((9*H*‐fluoren‐9‐yl)methoxy)(hydroxy)methylene)amino)‐*N*‐(1‐((4‐(8‐methoxy‐7‐((5‐((7‐methoxy‐2‐(4‐(4‐methylpiperazin‐1‐yl)phenyl)‐5‐oxo‐5,11a‐dihydro‐1*H*‐benzo[*e*]pyrrolo[1,2‐*a*][1,4]diazepin‐8‐yl)oxy)pentyl)oxy)‐10‐oxo‐3,3a,10,10a‐tetrahydrobenzo[*b*]cyclopenta[*e*]azepin‐2‐yl)phenyl)amino)‐1‐oxopropan‐2‐yl)‐3‐methylbutanimidic acid
	IV. 2‐((2‐amino‐1‐hydroxypropylidene)amino)‐*N*‐(2‐((di(1*H*‐imidazol‐2‐yl)methyl)imino)‐2‐hydroxyethyl)‐3‐phenylpropanimidic acid
	V*. N*‐(4‐guanidinobutyl)‐3‐(4‐hydroxyphenyl)acrylimidic acid
	VI. 2‐(acetoxymethyl)‐6‐(2‐(2‐((benzyloxy)(hydroxy)methylene)‐1‐(2‐(*tert*‐butoxy)‐2‐oxoethyl)hydrazineyl)ethyl)tetrahydro‐2*H*‐pyran‐3,4,5‐triyl triacetate
	VII. 1‐(6‐amino‐2‐((2‐((2‐amino‐1‐hydroxyethylidene)amino)‐1‐hydroxy‐3‐(1*H*‐imidazol‐5‐yl)propylidene)amino)hexanoyl)‐*N*‐(1‐oxo‐3‐phenylpropan‐2‐yl)pyrrolidine‐2‐carbimidic acid
*Lonicera japonica*	I. 5‐((2‐(3‐benzyl‐2‐oxopyrrolidin‐1‐yl)‐1‐hydroxyhexylidene)amino)‐4‐hydroxy‐*N*‐(1‐hydroxy‐3‐methyl‐1‐((pyridin‐2‐ylmethyl)imino)pentan‐2‐yl)‐2‐isopropyl‐7‐methyloctanimidic acid
	II. 5,5′‐((butane‐1,4‐diylbis(azanediyl))bis(2‐imino‐1,2‐dihydro‐9*H*‐purine‐6,9‐diyl))bis(2‐(hydroxymethyl)tetrahydrofuran‐3,4‐diol)
	III. 26‐amino‐4,7,10,13,16,19,22‐heptahydroxy‐8‐(hydroxymethyl)‐5,17‐diisobutyl‐24‐isoleucyl‐23‐isopropyl‐27‐methyl‐25‐oxo‐3,6,9,12,15,18,21,24‐octaazanonacosa‐3,6,9,12,15,18,21‐heptaenoic acid
	IV*. O*,*O*′‐(but‐2‐enedioyl)bis(*N*‐((4,5‐dihydro‐1*H*‐imidazol‐2‐yl)methyl)‐*N*‐(2‐(1‐methyl‐1*H*‐pyrazol‐4‐yl)phenyl)hydroxylamine)
	V. 2‐(1,3‐dimethyl‐2,6‐dioxo‐1,2,3,6‐tetrahydro‐7*H*‐purin‐7‐yl)‐*N*‐(6‐((hydroxymethylene)amino)‐4‐imino‐1,4‐dihydro‐1,3,5‐triazin‐2‐yl)acetimidic acid
	VI. 1,4‐bis(6‐hydroxy‐2‐imino‐2,3‐dihydro‐9*H*‐purin‐9‐yl)butane‐1,4‐diol
	VII*. N*‐(1‐((3‐(1‐indol‐3‐yl)‐1‐methoxy‐1‐oxopropan‐2‐yl)imino)‐1‐hydroxy‐3‐(1*H*‐indol‐3‐yl)propan‐2‐yl)‐3,5‐diguanidinobenzimidic acid
	VIII. 2‐oxo‐2‐(9,11,17‐trihydroxy‐10,13‐dimethyl‐3‐oxo‐2,3,6,7,8,9,10,11,12,13,14,15,16,17‐tetradecahydro‐1*H*‐cyclopenta[*a*]phenanthren‐17‐yl)ethyl hexanoate
	IX. 3‐(hydroxy(methylimino)methyl)‐6‐((1‐hydroxyhexadecylidene)amino)‐5‐oxoheptanoic acid
	X*. N*‐(1‐(1*H*‐imidazol‐5‐yl)‐3‐oxobutan‐2‐yl)‐2‐((1‐hydroxy‐2‐((1‐hydroxy‐2‐((1‐hydroxyethylidene)amino)‐3‐(1*H*‐imidazol‐5‐yl)propylidene)amino)‐3‐(1*H*‐imidazol‐5‐yl)propylidene)amino)‐3‐(1*H*‐imidazol‐5‐yl)propanimidic acid
	XI. 10‐(dimethylamino)‐6‐((4‐(dimethylamino)‐3‐hydroxy‐6‐methyltetrahydro‐2*H*‐pyran‐2‐yl)oxy)‐14‐ethyl‐7,12,13‐trihydroxy‐4‐((5‐hydroxy‐4‐methoxy‐4,6‐dimethyltetrahydro‐2*H*‐pyran‐2‐yl)oxy)‐3,5,7,9,11‐pentamethyl‐13‐((methylamino)methyl)oxacyclot etradecan‐2‐one
	XII. 5‐(hydroxy(phenylimino)methoxy)‐4‐(2‐(hydroxy(phenylimino)methoxy)ethyl)‐6‐methoxy‐2‐methyltetrahydro‐2*H*‐pyran‐3‐yl hydrogen (2‐methoxy‐6‐methylphenyl)carbonimidate
	XIII. 1‐(1‐(2‐((2‐((2‐amino‐1‐hydroxyethylidene)amino)‐1‐hydroxy‐3‐methylpentylidene)amino)‐5‐guanidinopentanoyl)pyrrolidin‐2‐yl)‐12‐benzyl‐9‐(3‐guanidinopropyl)‐1,4,7,10‐tetrahydroxy‐3‐isopropyl‐2,5,8,11‐tetraazatrideca‐1,4,7,10‐tetraen‐13‐oic acid
*Lonicera maackii*	I. 5‐((2‐(3‐benzyl‐2‐oxopyrrolidin‐1‐yl)‐1‐hydroxyhexylidene)amino)‐4‐hydroxy‐*N*‐(1‐hydroxy‐3‐methyl‐1‐((pyridin‐2‐ylmethyl)imino)pentan‐2‐yl)‐2‐isopropyl‐7‐methyloctanimidic acid
	II. 5,5′‐((butane‐1,4‐diylbis(azanediyl))bis(2‐imino‐1,2‐dihydro‐9*H*‐purine‐6,9‐diyl))bis(2‐(hydroxymethyl)tetrahydrofuran‐3,4‐diol)
	III. 26‐amino‐4,7,10,13,16,19,22‐heptahydroxy‐8‐(hydroxymethyl)‐5,17‐diisobutyl‐24‐isoleucyl‐23‐isopropyl‐27‐methyl‐25‐oxo‐3,6,9,12,15,18,21,24‐octaazanonacosa‐3,6,9,12,15,18,21‐heptaenoic acid
	IV*. O*,*O*′‐(but‐2‐enedioyl)bis(*N*‐((4,5‐dihydro‐1*H*‐imidazol‐2‐yl)methyl)‐*N*‐(2‐(1‐methyl‐1*H*‐pyrazol‐4‐yl)phenyl)hydroxylamine)
	V. 2‐(1,3‐dimethyl‐2,6‐dioxo‐1,2,3,6‐tetrahydro‐7*H*‐purin‐7‐yl)‐*N*‐(6‐((hydroxymethylene)amino)‐4‐imino‐1,4‐dihydro‐1,3,5‐triazin‐2‐yl)acetimidic acid
	VI. 1,4‐bis(6‐hydroxy‐2‐imino‐2,3‐dihydro‐9*H*‐purin‐9‐yl)butane‐1,4‐diol
	VII*. N*‐(1‐((3‐(1*H*‐indol‐3‐yl)‐1‐methoxy‐1‐oxopropan‐2‐yl)imino)‐1‐hydroxy‐3‐(1*H*‐indol‐3‐yl)propan‐2‐yl)‐3,5‐diguanidinobenzimidic acid
	VIII. 2‐oxo‐2‐(9,11,17‐trihydroxy‐10,13‐dimethyl‐3‐oxo‐2,3,6,7,8,9,10,11,12,13,14,15,16,17‐tetradecahydro‐1*H*‐cyclopenta[*a*]phenanthren‐17‐yl)ethyl hexanoate
	IX. 3‐(hydroxy(methylimino)methyl)‐6‐((1‐hydroxyhexadecylidene)amino)‐5‐oxoheptanoic acid
	X*. N*‐(1‐(1*H*‐imidazol‐5‐yl)‐3‐oxobutan‐2‐yl)‐2‐((1‐hydroxy‐2‐((1‐hydroxy‐2‐((1‐hydroxyethylidene)amino)‐3‐(1*H*‐imidazol‐5‐yl)propylidene)amino)‐3‐(1*H*‐imidazol‐5‐yl)propylidene)amino)‐3‐(1*H*‐imidazol‐5‐yl)propanimidic acid
	XI. 10‐(dimethylamino)‐6‐((4‐(dimethylamino)‐3‐hydroxy‐6‐methyltetrahydro‐2*H*‐pyran‐2‐yl)oxy)‐14‐ethyl‐7,12,13‐trihydroxy‐4‐((5‐hydroxy‐4‐methoxy‐4,6‐dimethyltetrahydro‐2*H*‐pyran‐2‐yl)oxy)‐3,5,7,9,11‐pentamethyl‐13‐((methylamino)methyl)oxacyclotetradecan‐2‐one
	XII. 5‐(hydroxy(phenylimino)methoxy)‐4‐(2‐(hydroxy(phenylimino)methoxy)ethyl)‐6‐methoxy‐2‐methyltetrahydro‐2*H*‐pyran‐3‐yl hydrogen (2‐methoxy‐6‐methylphenyl)carbonimidate
	XIII. 1‐(1‐(2‐((2‐((2‐amino‐1‐hydroxyethylidene)amino)‐1‐hydroxy‐3‐methylpentylidene)amino)‐5‐guanidinopentanoyl)pyrrolidin‐2‐yl)‐12‐benzyl‐9‐(3‐guanidinopropyl)‐1,4,7,10‐tetrahydroxy‐3‐isopropyl‐2,5,8,11‐tetraazatrideca‐1,4,7,10‐tetraen‐13‐oic acid
	XIV. 5‐hydroxy‐2‐(4‐hydroxy‐3‐((3,4,5‐trihydroxy‐6‐(hydroxymethyl)tetrahydro‐2*H*‐pyran‐2‐yl)oxy)phenyl)‐7‐methoxy‐4*H*‐chromen‐4‐one
	XV. 1‐(3,4‐dihydroxy‐5‐((phosphonooxy)methyl)tetrahydrofuran‐2‐yl)‐5‐((hydroxymethylene)amino)‐*N*‐methyl‐1*H*‐imidazole‐4‐carbimidic acid
	XVI. 4‐hydroxy‐2‐((3,4,5‐trihydroxy‐6‐(4‐nitrophenoxy)tetrahydro‐2*H*‐pyran‐2‐yl)methoxy)‐6‐(1,2,3‐trihydroxypropyl)tetrahydro‐2*H*‐pyran‐2‐carboxylic acid
*Microstegium vimineum*	I. (6‐((3,12‐dihydroxy‐4,4,8,10,14‐pentamethyl‐17‐(6‐methylhepta‐2,5‐dien‐2‐yl)hexadecahydro‐1*H*‐cyclopenta[*a*]phenanthren‐6‐yl)oxy)‐3,4,5‐trihydroxytetrahydro‐2*H*‐pyran‐2‐yl)methyl acetate
	II. (butane‐1,4‐diylbis(7,9‐diethyl‐2‐hydroxy‐6,7,9‐trimethyl‐4‐oxo‐1,3,8‐triazaspiro[4.5]dec‐1‐ene‐3,8‐diyl))bis(ethane‐2,1‐diyl) dibenzoate
	III. 10‐(dimethylamino)‐6‐((4‐(dimethylamino)‐3‐hydroxy‐6‐methyltetrahydro‐2*H*‐pyran‐2‐yl)oxy)‐14‐ethyl‐7,12,13‐trihydroxy‐4‐((5‐hydroxy‐4‐methoxy‐4,6‐dimethyltetrahydro‐2*H*‐pyran‐2‐yl)oxy)‐3,5,7,9,11‐pentamethyl‐13‐((methylamino)methyl)oxacyclotetradecan‐2‐one
	IV. 26,30‐diamino‐2‐(4‐aminobutyl)‐4,7,10,13,16,19,22,25‐octahydroxy‐8‐(3‐hydroxy‐3‐iminopropyl)‐11,23‐diisobutyl‐5,14‐dimethyl‐3,6,9,12,15,18,21,24‐octaazatriaconta‐3,6,9,12,15,18,21,24‐octaenoic acid
	V. 2‐((4,5‐dihydroxy‐2‐((7‐hydroxy‐3‐(2‐hydroxypropan‐2‐yl)‐5a,5b,8,8,11a,13b‐hexamethyl‐4‐((3,4,5‐trihydroxy‐6‐methyltetrahydro‐2*H*‐pyran‐2‐yl)oxy)icosahydro‐1 *H*‐cyclopenta[*a*]chrysen‐9‐yl)oxy)tetrahydro‐2*H*‐pyran‐3‐yl)oxy)‐6‐methyltetrahydro‐2*H*‐pyran‐3,4,5‐triol
	VI. 5‐((2‐ethyl‐5‐guanidino‐1‐hydroxypentylidene)amino)‐8‐guanidino‐2‐(3‐guanidinopropyl)‐*N*‐(7‐(hydroxy((1‐hydroxy‐1‐iminopropan‐2‐yl)imino)methyl)‐2,9‐dimethyl‐5‐oxodecan‐4‐yl)‐4‐oxooctanimidic acid
	VII. 3,6,9,11,14,17,20‐heptahydroxy‐4,12‐diisobutyl‐15,18‐diisopropyl‐7‐methyl‐5,8,13,16,19‐pentaazahexacosa‐5,8,13,16,19‐pentaenoic acid
	VIII. 2,2,6,6‐tetramethyl‐1‐(2‐((4‐(4‐pentylcyclohexyl)benzoyl)oxy)ethyl)piperidin‐4‐yl 4‐(4‐pentylcyclohexyl)benzoate
	IX.‐(1,17‐diamino‐9‐(3‐guanidinopropyl)‐8,11‐dihydroxy‐6‐(hydroxy(imino)methyl)‐1,17‐diimino‐2,7,10,16‐tetraazaheptadeca‐7,10‐dien‐12‐yl)tetradecanimidic acid
*Paulownia tomentosa*	I. *N*‐(1‐methyl‐5‐((1‐methyl‐5‐((1‐methyl‐5‐((2‐morpholinoethyl)carbamoyl)‐1*H*‐pyrrol‐3‐yl)carbamoyl)‐1*H*‐pyrrol‐3‐yl)carbamoyl)‐1*H*‐pyrrol‐3‐yl)‐1,6‐naphthyridine‐2‐carboxamide
	II. 4‐(6‐(4‐(2‐(acryloyloxy)ethoxy)phenyl)‐5‐(4‐(3‐(acryloyloxy)propyl)phenyl)naphthalen‐2‐yl)benzyl acrylate
	III*. N*‐(3‐cyclohexyl‐1‐((5‐guanidino‐1‐oxopentan‐2‐yl)imino)‐1‐hydroxypropan‐2‐yl)‐2‐((1‐hydroxyethylidene)amino)‐4‐methylpentanimidic acid
	IV. 6‐amino‐*N*‐(6‐amino‐1‐((6‐amino‐1‐hydroxy‐1‐((1‐hydroxy‐3‐methyl‐1‐((3‐methylbutan‐2‐yl)imino)butan‐2‐yl)imino)hexan‐2‐yl)imino)‐1‐hydroxyhexan‐2‐yl)‐2‐((1,4,7,10,13‐pentahydroxy‐2‐(hydroxymethyl)‐3,6,9,12‐tetraazatetradeca‐3,6,9,12‐tetraen‐1‐ylidene)amino)hexanimidic acid
	V. 4‐benzoyl‐*N*,1‐bis(2,4‐dinitrophenyl)‐5‐phenyl‐1*H*‐pyrazole‐3‐carbohydrazonic acid
	VI. 9‐(7‐(6‐(1‐(allyloxy)‐1‐oxobutan‐2‐yl)‐3‐methyltetrahydro‐2*H*‐pyran‐2‐yl)‐6‐hydroxy‐5‐methyl‐4‐oxooctan‐3‐yl)‐2‐(5‐ethyl‐5‐hydroxy‐6‐methyltetrahydro‐2*H*‐pyran‐2‐yl)‐2,10,12‐trimethyl‐1,6,8‐trioxadispiro[4.1.5^7^.3^5^]pentadec‐13‐en‐15‐yl 1*H*‐imidazole‐1‐carboxylate
	VII*. N*‐(2‐(2‐(2‐(2‐(5‐cyclobutyl‐4‐methyl‐1,2,3‐triazolidin‐1‐yl)ethoxy)ethoxy)ethoxy)ethyl)‐6‐(2‐hydroxy‐4‐methyl‐4,5‐dihydro‐1*H*‐imidazol‐5‐yl)hexanimidic acid
*Persicaria perfoliata*	I. 6‐(4‐((((7‐(benzyloxy)‐1*H*‐benzo[*d*]imidazol‐5‐yl)imino)(hydroxy)methoxy)methyl)‐2‐nitrophenoxy)‐3,4,5‐trihydroxytetrahydro‐2*H*‐pyran‐2‐carboxylic acid
	II. 6‐((6‐((6‐((6‐(3‐(dimethylamino)phenoxy)‐4,5‐dihydroxy‐2‐(hydroxymethyl)tetrahydro‐2*H*‐pyran‐3‐yl)oxy)‐4,5‐dihydroxy‐2‐(hydroxymethyl)tetrahydro‐2*H*‐pyran‐3‐yl)oxy)‐4,5‐dihydroxy‐2‐methyltetrahydro‐2*H*‐pyran‐3‐yl)amino)‐4‐(hydroxymethyl)cyclohex‐4‐ene‐1,2,3‐triol
	III. 10‐((4,5‐dihydroxy‐3‐((3,4,5‐trihydroxy‐6‐methyltetrahydro‐2*H*‐pyran‐2‐yl)oxy)tetrahydro‐2*H*‐pyran‐2‐yl)oxy)‐2‐(hydroxymethyl)‐2,6a,6b,9,9,12a‐hexamethyl‐1,3,4,5,6,6a,6b,7,8,8a,9,10,11,12,12a,12b,13,14b‐octadecahydropicene‐4a(2*H*)‐carboxylic acid
	IV*. N*‐(1‐hydroxy‐3‐((3,4,5‐trihydroxy‐6‐methyltetrahydro‐2*H*‐pyran‐2‐yl)oxy)‐1‐(undecylimino)propan‐2‐yl)dodecanimidic acid
	V*. N* ^1^‐(2‐(diethylamino)ethyl)‐*N* ^2^,*N* ^2^‐diethyl‐*N* ^1^‐(4‐(1‐ethyl‐1*H*‐imidazo[4,5‐*b*]pyridin‐2‐yl)phenyl)ethane‐1,2‐diamine
	VI. 2,2,6a,6b,9,9,12a‐heptamethyl‐10‐((3,4,5‐trihydroxy‐6‐(hydroxymethyl)tetrahydro‐2*H*‐pyran‐2‐yl)oxy)‐1,3,4,5,6,6a,6b,7,8,8a,9,10,11,12,12a,12b,13,14b‐octadecahydropicene‐4a(2*H*)‐carboxylic acid
	VII. 11‐((*tert*‐butoxy(hydroxy)methylene)amino)undecanoic anhydride
	VIII*. N*‐(*sec*‐butyl)‐*N*′‐butyl‐*N*‐((5‐(4‐ethylpiperazin‐1‐yl)‐3‐methyl‐1‐phenyl‐1*H*‐pyrazol‐4‐yl)methyl)carbamimidic acid
	IX. 2‐(((1‐(2‐(((1‐(2‐((1,5‐dihydroxy‐2‐((hydroxy(pyrrolidin‐2‐yl)methylene)amino)‐5‐iminopentylidene)amino)‐5‐hydroxy‐5‐iminopentanoyl)pyrrolidin‐2‐yl)(hydroxy)methylene)amino)‐3‐phenylpropanoyl)pyrrolidin‐2‐yl)(hydroxy)methylene)amino)‐5‐hydroxy‐5‐iminopentanoic acid
	X. 6‐((1‐cyclobutylethyl)amino)‐8‐cyclohexyl‐7‐((4‐methylcyclohexyl)methyl)‐7*H*‐purine‐2‐carboxamide
*Pueraria montana*	I. methyl 3‐(5‐((3‐(1‐hydroxyethyl)‐4‐methyl‐5‐oxopyrrolidin‐2‐ylidene)methyl)‐2‐((3‐(3‐methoxy‐3‐oxopropyl)‐4‐methyl‐5‐((4‐methyl‐2‐oxo‐3‐vinyl‐2*H*‐pyrrol‐5‐yl)methylene)‐1,5‐dihydro‐2*H*‐pyrrol‐2‐ylidene)methyl)‐4‐methyl‐1*H*‐pyrrol‐3‐yl)propanoate
	II. 2,4‐bis((2,4‐dinitrophenyl)amino)cyclopentan‐1‐ol
	III. 2‐(3,4‐dihydroxyphenyl)‐5‐hydroxy‐7‐((3,4,5‐trihydroxy‐6‐(((3,4,5‐trihydroxy‐6‐methyltetrahydro‐2*H*‐pyran‐2‐yl)oxy)methyl)tetrahydro‐2*H*‐pyran‐2‐yl)oxy)‐4*H*‐chromen‐4‐one
	IV. tridecane‐1,4,7,10,13‐pentaamine
	V. 6‐hydroxy‐*N*‐(1‐hydroxy‐1‐((1‐hydroxy‐1‐((4‐methyl‐1‐((4‐nitrophenyl)amino)‐1‐oxopentan‐2‐yl)imino)‐3‐phenylpropan‐2‐yl)imino)propan‐2‐yl)‐2‐methyl‐3‐oxo‐2,3,4,5‐tetrahydropyridine‐2‐carbimidic acid
	VI*. N* ^1^,*N* ^4^,*N* ^7^,*N* ^10^‐tetra(pyridin‐4(1*H*)‐ylidene)‐1,4,7,10‐tetraazacyclododecane‐1,4,7,10‐tetracarboxamide
	VII*. O*,*O*′‐(but‐2‐enedioyl)bis(*N*‐((4,5‐dihydro‐1*H*‐imidazol‐2‐yl)methyl)‐*N*‐(2‐(1‐methyl‐1*H*‐pyrazol‐4‐yl)phenyl)hydroxylamine)
*Rosa multiflora*	I. propane‐2,2‐diylbis(cyclohexane‐4,1‐diyl) bis(hydrogen butylcarbonimidate)
	II. 10‐((1‐carboxy‐2‐phenylethyl)(methyl)carbamoyl)‐3,12,15‐trihydroxy‐16‐((1‐hydroxyethylidene)amino)‐5‐imino‐13‐methyl‐2,4,6,11,14‐pentaazaoctadeca‐2,11,14‐trien‐18‐oic acid
	III. 2‐((hydroxy(prop‐2‐yn‐1‐yloxy)methylene)amino)‐*N*‐(1‐oxo‐1‐(pentyloxy)hexan‐2‐yl)hexanimidic acid
	IV*. N*‐(4‐(5‐((5‐((5‐((3‐(dimethylamino)propyl)carbamoyl)‐1‐methyl‐1*H*‐pyrrol‐3‐yl)carbamoyl)‐1‐methyl‐1*H*‐pyrrol‐3‐yl)carbamoyl)‐1‐methyl‐1*H*‐pyrrol‐3‐yl)phenyl)acetimidic acid
	V. 4‐(6‐(4‐(2‐(acryloyloxy)ethoxy)phenyl)‐5‐(4‐(3‐(acryloyloxy)propyl)phenyl)naphthalen‐2‐yl)benzyl acrylate
	VI. 6‐amino‐*N*‐(6‐amino‐1‐((6‐amino‐1‐hydroxy‐1‐((1‐hydroxy‐3‐methyl‐1‐((3‐methylbutan‐2‐yl)imino)butan‐2‐yl)imino)hexan‐2‐yl)imino)‐1‐hydroxyhexan‐2‐yl)‐2‐((1,4,7,10,13‐pentahydroxy‐2‐(hydroxymethyl)‐3,6,9,12‐tetraazatetradeca‐3,6,9,12‐tetraen‐1‐ylidene)amino)hexanimidic acid
	VII. 4‐amino‐*N*‐(2‐((2‐((2‐((5‐(methoxycarbonyl)‐1‐methyl‐1*H*‐pyrrol‐3‐yl)carbamoyl)‐1‐methyl‐1*H*‐imidazol‐4‐yl)carbamoyl)‐1‐methyl‐1*H*‐imidazol‐4‐yl)carbamoyl)‐1‐methyl‐1*H*‐imidazol‐4‐yl)butanimidic acid
*Rubus phoenicolasius*	I. 2‐((1,3‐dihydroxy‐2‐((1‐hydroxy‐2‐((1‐hydroxyethylidene)amino)‐3‐methylpentylidene)amino)propylidene)amino)‐*N*‐(1‐((1‐(ethylimino)‐5‐guanidino‐1‐hydroxypentan‐2‐yl)imino)‐1‐hydroxy‐3‐methylpentan‐2‐yl)pentanebis(imidic) acid
	II. propyl 3‐((5‐(aminomethyl)‐5‐hydroxy‐4‐methoxy‐4,6‐dimethyltetrahydro‐2*H*‐pyran‐2‐yl)oxy)‐9‐((3,4‐dihydroxy‐4‐methylpentan‐2‐yl)(methyl)amino)‐5‐((4‐(dimethylamino)‐3‐hydroxy‐6‐methyltetrahydro‐2*H*‐pyran‐2‐yl)oxy)‐6‐hydroxy‐2,4,6,8‐tetramethylnonanoate
	III. 6‐amino‐2‐((6‐amino‐2‐((6‐amino‐2‐((2,6‐diamino‐1‐hydroxyhexylidene)amino)‐1‐hydroxyhexylidene)amino)‐1‐hydroxyhexylidene)amino)hexanoic acid
	IV. 2‐(1‐(4‐amino‐5‐phenyl‐7*H*‐pyrrolo[2,3‐*d*]pyrimidin‐7‐yl)‐2‐hydroxypropoxy)‐3‐hydroxypropyl dihydrogen phosphate
	V*. N*‐(1,17‐diamino‐9‐(3‐guanidinopropyl)‐8,11‐dihydroxy‐6‐(hydroxy(imino)methyl)‐1,17‐diimino‐2,7,10,16‐tetraazaheptadeca‐7,10‐dien‐12‐yl)tetradecanimidic acid
	VI. di‐*tert*‐pentyl dodecane‐1,12‐diylbis(hydrogen carbonimidate)
	VII*. N*‐(3,3‐dimethyl‐1‐(2‐(((3‐methyl‐1‐phenyl‐1*H*‐pyrazol‐5‐yl)amino)methyl)pyrrolidin‐1‐yl)‐1‐oxobutan‐2‐yl)‐2‐(methylamino)propanimidic acid
	VIII. ethyl 4‐((6‐(diisopentylcarbamoyl)‐1‐(3‐(methyl(pentyl)amino)propyl)‐1,3‐dihydro‐2*H*‐benzo[*d*]imidazol‐2‐ylidene)amino)piperidine‐1‐carboxylate
	IX*. N*‐(3‐((4‐(dimethylamino)‐2‐((2‐ethyl‐3,4,10,13‐tetrahydroxy‐3,5,6,8,10,12,14‐heptamethyl‐15‐oxo‐1‐oxa‐6‐azacyclopentadecan‐11‐yl)oxy)‐6‐methyltetrahydro‐2*H*‐pyran‐3‐yl)oxy)propyl)‐2‐hydroxyacetimidic acid
	X. 20‐amino‐4,7,10,13,16,19‐hexahydroxy‐2,5,17‐tris(1‐hydroxyethyl)‐11‐(hydroxymethyl)‐8,14‐dimethyl‐3,6,9,12,15,18‐hexaazaicosa‐3,6,9,12,15,18‐hexaenoic acid

Note

Compounds were identified by matching isotopic patterns and MS/MS spectra to the PubChem MS database using Sirius4 (Dürhkop et al. 2019).

The 15 invasive plant species had 9,143 compounds not found among the 46 native species, of which the most abundant are presented in Figure 2. The native species had 30,774 compounds not found in invasive species. Just 1,084 compounds were found in both invasive and native species. In many instances, compounds unique to either invasive or native plant species comprised subnetworks of structurally similar compounds (Figure 1). Such clusters of structurally similar compounds may represent structural precursors or alternative products from shared metabolic pathways.

**FIGURE 3:**
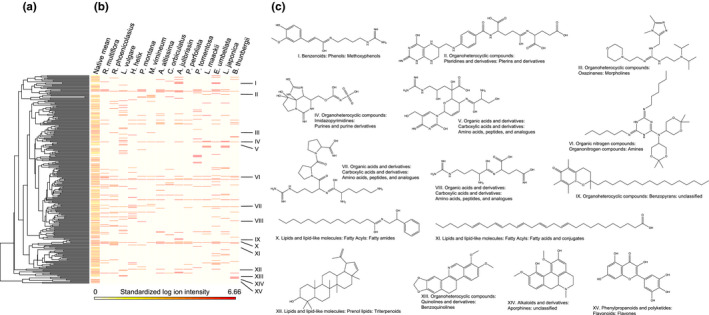
Classification of abundant compounds in native and invasive plant species. Panel a presents a phylogeny‐like tree that reflects the structural similarity among compounds, generated using Qemistree (Tripathi et al., 2020). Panel b presents a heat map representing the mean log ion intensity of compounds in 46 native species, weighted by species abundances at SERC (column 1) and the log ion intensity of compounds in each of 15 invasive species (columns 2–15). Invasive species are ranked by CSCS_native_, their chemical similarity to the native community (Figure 4). Panel c presents structures and chemotaxonomic classifications of 15 compounds using ClassyFire (Feunang et al., 2016)

The most chemically novel invasive species were *B. thunbergii*, *L. japonica* and *Lonicera maackii*, *Elaegnus umbellate*, *Paulownia tomentosa*, *P. perfoliata*, *Albizia julibrissin*, *Celastrus orbiculatus*, and *Ailanthus altissima* (Table 1). Compounds unique to *B. thunbergii* included a benzylisoquinoline alkaloid similar in structure to berberine (Figure 2cVIII, Figure 3cXIII), which occurs in other species of *Berberis* (Stermitz, Lorenz, Tawara, Zenewicz, & Lewis, 2000). Other compounds that were uniquely found among invasive species included pterins (*L. japonica* and *L. maackii*), methoxyphenols (*A. julibrissin* and *Elaegnus umbellata*), and peptides (*Ligustrum vulgare* and *P. montana*) (Figure 3). A dendrogram presenting the structural relationships and classifications of 267 abundant compounds generated using Qemistree (Tripathi et al., 2020) and a heat map presenting the occurrence of these compounds in native and invasive species is presented in Figure 3.

**FIGURE 4:**
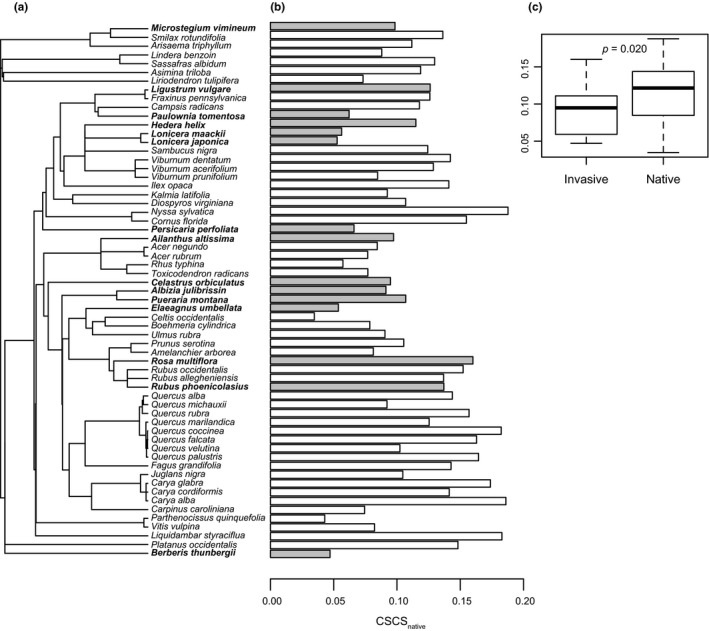
Invasive plant species are less chemically similar than native species to the native flora of SERC, Maryland. Panel a presents phylogenetic relationships among invasive and native plant species. Panel b presents the mean abundance‐weighted chemical structural‐compositional similarity of each species to native plant species (CSCS_native_) in the SERC Forest Dynamics Plot. White and gray bars represent native and invasive species, respectively. Phylogenetic signal in CSCS_native_ is low (*K* = 0.05, *p* = .48). Panel c presents the distribution of CSCS_native_ for invasive and native plant species in a boxplot (generalized least squares analysis of variance, *F*_1,59_ = 5.760, *p* = .020)

Invasive plant species were less chemically similar than were native plant species to the native flora of SERC (GLS ANOVA *F*_1,59_ = 5,760, *p* = .020; Figure 4, Table 5a;). The mean CSCS_native_ ± 1 standard error was 0.091 ± 0.009 and 0.118 ± 0.006 for invasive and native species, respectively (Table 5a), and CSCS_native_ exhibited little phylogenetic signal in the community as a whole (*K* = 0.05*, p* = .48). Invasive species were not less preferred by either the Japanese beetle (ANOVA *F*_1,20_ = 1.878, dof = 20, *p* = .186; Table 5b) or the woolly bear caterpillar (ANOVA *F*_1,26_ = 0.169, dof = 26, *p* = .685; Table 5c). Likewise, invasive plant species did not exhibit lower browsing rates by white‐tailed deer (weighted PGLS ANOVA *F*_1,57_ = 0.0735, dof = 57, *p* = .787; Table 5d). The mean proportion of individuals browsed, weighted by the number of individuals observed per species, was 0.536 and 0.522 for invasive and native species, respectively. A MANOVA of CSCS_native_ and seven functional traits indicated that invasive species exhibited greater foliar N, P, and water content, and lower CSCS_native_ and leaf toughness than native species (Table 5e).

**TABLE 5:** Mean (±*SE*) abundance‐weighted values for CSCS chemical similarity relative to native species (CSCS_native_: a), herbivore palatability (b‐d), and seven functional traits (e) for native and invasive species at SERC, Maryland

(a)	Trait	Native	Invasive	ANOVA *F*_1,58_
60 Species^a^	Mean CSCS_native_	0.118 ± 0.006	0.091 ± 0.009	**5.760** *
**(b)**	**Trait**	**Native**	**Invasive**	**ANOVA *F*_1,20_**
22 Species^a^	Japanese Beetle Preference	0.456 ± 0.061	0.344 ± 0.055	1.878
**(c)**	**Trait**	**Native**	**Invasive**	**ANOVA *F*_1,26_**
28 Species^a^	Woolly Bear Caterpillar Preference	0.447 ± 0.052	0.484 ± 0.080	0.169
**(d)**	**Trait**	**Native**	**Invasive**	**ANOVA *F*_1,57_**
59 Species^a^	Proportion Browsed	0.522	0.536	0.073
**(e)**	**Trait**	**Native**	**Invasive**	**MANOVA *F*_1,44_**
48 Species^a^	Mean CSCS_native_	0.123 ± 0.007	0.092 ± 0.010	**6.870** *
	% Water	65 ± 2	71 ± 2	**4.000** *
	SLA (g/cm^2^)	350 ± 17	391 ± 57	0.821
	Toughness (*N*)	2.1 ± 0.2	1.3 ± 0.2	**4.131** *
	Trichomes (cm^−2^) ^b^	120 ± 28	2,452 ± 2,268	0.646
	% C	45.7 ± 0.3	45.3 ± 0.5	0.476
	% N	2.13 ± 0.07	2.9 ± 0.3	**14.04** ***
	% P	0.19 ± 0.01	0.28 ± 0.03	**13.531** ***

Note

Blomberg's *K* and test for phylogenetic signal in the residual error (a): *K* = 0.046, *p* = .529. Full phylogenetic MANOVA (c) *F*_1,44_ = 3.376, *p* = .005, *p*_given phylogeny_ = .020.

^a^ The ANOVAs compared the 46 native and 15 invasive species illustrated in Figure 1 (a), 11 native and 11 invasive species assayed for Japanese beetle diet preference (b), 16 native and 12 invasive species for which woolly bear caterpillar diet preference results were available (Lind & Parker, 2010; c), and 44 native and 15 invasive species for which deer browse was recorded. (d) The MANOVA compared 33 native and 13 invasive species for which functional trait data were also available (Lind & Parker, 2010; Table 2; e).

^b^ log transformed for analysis.

* *p* < .05;

** *p* < .01;

*** *p* < .001.

**FIGURE 5:**
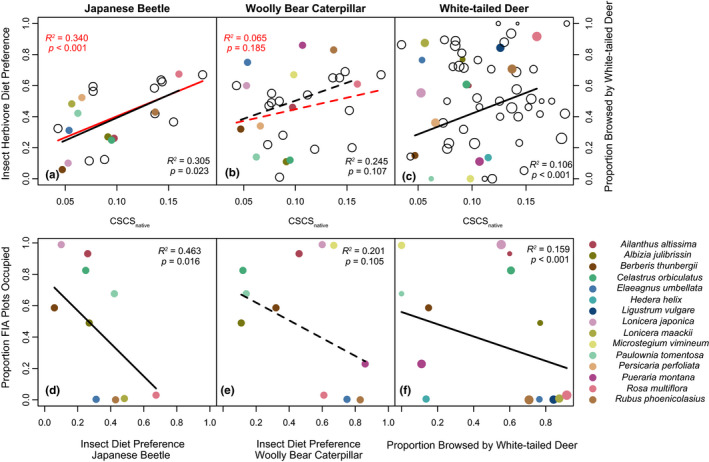
Evaluation of the novel weapons and enemy release hypotheses for three locally abundant herbivores. In panels a–c, red lines indicate regressions performed for all plant species, black lines indicate regressions performed for only invasive species. In panels d–f, only invasive species were considered (black lines). Solid lines indicate significant beta regressions; dashed lines indicate insignificant trends. Open symbols represent native plant species; colored symbols represent invasive species, as indicated at right. Symbol size is proportional to species log abundance for abundance‐weighted regressions in panels c and f. When considered together, both insect herbivores preferred less chemically novel species in the SERC plant community (all local species: *R* ^2^ = .225, *p* = .014; panels a,b) and species preferred less by insect herbivores occupied a greater proportion of FIA “invasive species” survey plots in the Atlantic coastal plain (*R* ^2^ = .325, *p* = .022; panels d,e)

Plant species that were chemically distinct relative to the native community at SERC were less preferred by locally common herbivores. The Japanese beetle significantly preferred invasive plant species that were more chemically similar to the native flora at SERC (i.e., species with large CSCS_native_; *R* ^2^ = .305, *p* = .023; Figure 5a; see Blomberg's *K* statistic for phylogenetic signal in the residual error for this and all subsequent regression models in Table 3). Likewise, among all native and invasive plant species combined, the Japanese beetle preferred those that were more chemically similar to the native plant community at SERC (*R* ^2^ = .340, *p* = .001; Figure 5a).

In contrast with the Japanese beetle, the diet preference of the native woolly bear caterpillar was not related to CSCS_native_ for either all plants (*R* ^2^ = .065, *p* = .185; Figure 5b) or invasive species considered alone (*R* ^2^ = .122, *p* = .245; Figure 5b). When both insect herbivores were considered together in a combined model, both herbivores preferred to feed on invasive species that were more chemically similar to the native flora at SERC (*R* ^2^ = .247, *p* = .018). Likewise, when native and invasive host plants were considered together, both herbivores preferred host plants that were more chemically similar to the native plant community (*R* ^2^ = .225, *p* = .014).

When both native and invasive species were considered together, there was no relationship between chemical novelty and browse by white‐tailed deer (Figure 5c). The residual error of the beta regression exhibited low, but significant phylogenetic signal (*K* = 0.172, *p* = .012; Table 3), and a phylogenetic generalized least squares regression did not support the relationship (*p* = .324). In contrast, among only invasive species, chemically novel invasive species experienced lower rates of deer browse (*R* ^2^ = .106, *p* < .001; Figure 5c).

**FIGURE 6:**
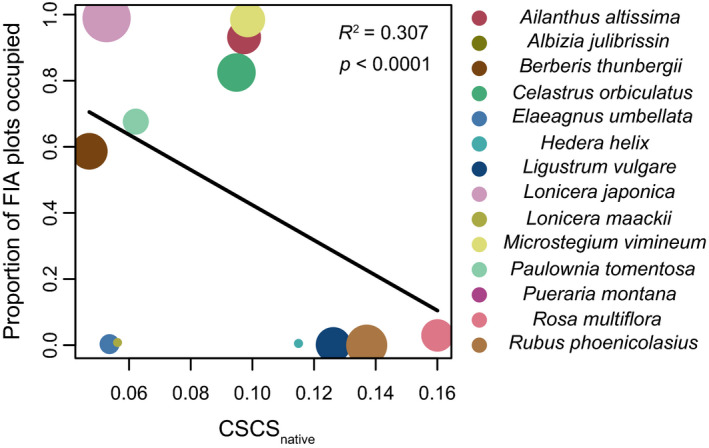
Invasive species that were more chemically distinct (smaller CSCS_native_) were found in a greater proportion of FIA “invasive species” survey plots across the Atlantic coastal plain. The regression was weighted by abundance at SERC. The area of each point reflects the log abundance of the species. Symbol colors represent invasive species, as indicated at right

The frequency of invasion at the regional scale was related to variation among invasive species in insect herbivore diet preference, white‐tailed deer browse, and chemical distinctiveness. Invasive species that were preferred less by the Japanese beetle occupied a greater proportion of FIA “invasive species” plots in the Atlantic coastal plain (*R* ^2^ = .463, *p* = .016; Figure 5d). Woolly bear diet preference was not related to the proportion of FIA plots occupied by invasive species (*R* ^2^ = .201, *p* = .105; Figure 5e); however, invasive species avoided by both insect herbivores occupied a greater proportion of plots (*R* ^2^ = .325, *p* = .022). Likewise, invasive plant species avoided by white‐tailed deer occupied a greater proportion of FIA plots in the coastal plain (*R* ^2^ = .159, *p* < .001; Figure 5f). Finally, we observed a direct relationship between invasion frequency and chemical novelty, as invasive species that were more chemically distinct relative to the native flora at SERC occupied a greater proportion of FIA “invasive species” plots in the Atlantic coastal plain (*R* ^2^ = .307, *p* < .001; Figure 6).

## 4 DISCUSSION

The enemy release hypothesis posits that biological invasions are driven by escape from naïve native herbivores in the introduced range (Keane & Crawley, 2002). The novel weapons hypothesis attributes this release to novel secondary metabolites that deter naïve herbivores in the introduced range with which the invaders do not share a coevolutionary history (Cappuccino & Arnason, 2006; Lind & Parker, 2010; Verhoeven et al., 2009). The overwhelming diversity of plant secondary metabolites has precluded a test of the enemy release and novel weapons hypotheses at the intersection of complex plant metabolomes and diverse ecological communities. We overcame the challenge of chemical diversity by taking advantage of mass spectrometry bioinformatics techniques that assemble spectra from unknown compounds into molecular networks to quantify the structural similarity of compounds to each other and to annotated spectra from databases (Dührkop et al., 2019; Tripathi et al., 2020; Wang et al., 2016). Molecular networks representing foliar metabolomes indicate that 15 invasive species are less similar chemically than are 46 native species to the native flora recorded at the Smithsonian Environmental Research Center in Edgewater, Maryland. Furthermore, among the 15 invasive species, the more chemically novel species exhibit reduced palatability to insect herbivores and reduced whitetail deer herbivory, and are more widespread in 2,505 FIA invasive species census plots in the Atlantic coastal plain of the eastern United States. These results provide evidence in support of the predictions of the enemy release and novel weapons hypotheses and suggest that by facilitating release from herbivory by the common herbivores in eastern North America, chemical novelty facilitates biological invasions for many invasive plant species.

Invasive species are less similar chemically than native species to the native plant community at SERC, but not dramatically so (Figure 4a–c, Table 5). Likewise, an examination of the chemical classifications of the 267 most abundant compounds indicates that a few invasive species exhibit benzoquinolines, methoxyphenols, pterins, and peptides that are novel to the plant community at SERC, yet many more compounds are shared among natives and invasives, or found exclusively among the native plants (Figure 3). Such compounds include amines, aporphines, flavones, morpholines, purine derivatives, and triterpenoids (Figure 3). Further, invasive species as a group are not avoided by Japanese beetles, wooly bear caterpillars, or white‐tailed deer (Table 5b–d). Together, these observations suggest that not all invasions are driven by chemical novelty relative to the native plant community. In pursuit of a better understanding of the relationship between chemical novelty and invasion, we consider the variation in chemical novelty among the invasive species.

Variation in chemical distinctiveness among invasive species is related to insect and ungulate herbivory and invasion frequency in a manner that suggests herbivore release favors successful invasions. Chemically distinct invasive species (low CSCS_native_) tend to exhibit reduced insect herbivore diet preference (Figure 5a,b) and to experience reduced browsing by white‐tailed deer (Figure 5c). Furthermore, species that are disliked by insect herbivores (Figure 4d,e) and experience less browsing by white‐tailed deer (Figure 5f) occupy a greater proportion of FIA plots in the Atlantic coastal plain. Finally, invasive species that are chemically novel compared to native species occupy a greater proportion of FIA plots distributed across the Atlantic coastal plain (Figure 6). Collectively, these observations suggest that more successful invaders tend to be chemically dissimilar from the native flora, avoid herbivory by insect herbivores and white‐tailed deer, and are thus promoted by herbivores. In sum, our results suggest that chemical novelty favors biological invasions for some species by facilitating ecological release from herbivory by common insect herbivores and by the dominant large mammalian herbivore in eastern North America.

Invasive trees *A. altissima* and *P. tomentosa*, the invasive shrub *B. thunbergii*, and the invasive grass *M. vimineum* all appear chemically novel, avoided by deer and insect herbivores, and widely invasive in the Atlantic coastal plain (Figures 5, 6) and therefore represent likely examples of invasions facilitated by novel antiherbivore chemical defenses. The invasive vine *C. orbiculatus* appears susceptible to deer herbivory (Figure 5c), but may experience release from insect herbivores (Figure 5a,b). These species exhibit alkaloids and phenolic compounds not found in the native plant community (Figures 2, 3). In the case of *M. vimineum*, high foliar silica content likely contributes to avoidance by deer (Abrams & Johnson, 2012), though silicate phytoliths would not be detected using our metabolomics methods.

In addition to lower CSCS_native_, invasive plant species exhibited greater foliar N, P, and water content and lower leaf toughness than native species (Table 5e). These traits are widely associated with greater growth rates in a global tradeoff between potential growth rates and stress tolerance (Reich, 2014). For some of the species considered here, such as *L. japonica* and *R. multiflora*, high growth rates and competitive ability may drive their invasion success (Averill et al., 2018; Parker et al., 2011). For others, foliar traits that facilitate quick return on resource investment and high maximum growth rates may allow them to take advantage of the enemy release conferred by their novel secondary chemistry.

In sum, our results suggest that not all invasive species are successful due to novel chemical defenses. However, invasive species exhibited patterns of chemical novelty and avoidance of insect and mammalian herbivores consistent with the novel weapons and enemy release hypotheses, suggesting that these mechanisms contribute to many successful invasions.

### 4.1 Implications for invasion mechanisms and effects

Can we reconcile mammalian browsing behavior with the novel weapons mechanism of ecological release? Mammalian herbivores tend to consume more plant species than do insect herbivores and are able to detoxify a broad range of plant chemical defenses by foraging selectively so as to minimize consumption of any single toxin (Foley & Moore, 2005). Hence, Verhoeven et al. (2009) proposed that, whereas insect herbivores might be deterred by novel secondary metabolites, mammalian herbivores may preferentially browse plants with locally novel secondary metabolites to avoid locally common toxins and thereby spread the toxin load over different detoxification metabolic pathways (Marsh, Wallis, McLean, Sorensen,& Foley, 2006; Verhoeven et al., 2009). Chemically, novel introduced species would therefore be suppressed by mammalian herbivores (Verhoeven et al., 2009). The invasive species considered here directly contradict this prediction with respect to white‐tailed deer (Figure 5c). However, we considered only introduced species recognized as successful invaders. Unsuccessful invaders may include species that are chemically redundant with the native flora, as well as species that are chemically novel but suppressed by mammals such as deer (Verhoeven et al., 2009). Our results suggest that many successful invaders exhibit novel chemistry that is effective against insect herbivores and white‐tailed deer (Figure 5).

Biological invasions are often invoked to explain biodiversity losses in native plant communities (Didham, Tylianakis, Hutchison, Ewers, & Gemmell, 2005). However, plant invasions may not be a direct cause of native species declines but rather may be artifactually correlated with reductions in native diversity driven by other ecological changes (Didham et al., 2005). White‐tailed deer populations are at the highest levels ever recorded in eastern North America as a result of anthropogenic habitat modification and predator extirpation (Royo, Collins, Adams, Kirschbaum, & Carson, 2010; Pendergast, Hanlon, Long, Royo, & Carson, 2016). Herbivory by overabundant deer is most likely directly responsible for declines of many native species (Royo *et al*., 2010; Pendergast *et al*., 2016). Our results suggest that overabundant deer also facilitate invasions by chemically novel species that are avoided (Figure 5c,f); they may also promote the ecological success of ruderal, browse‐tolerant, or fast‐growing species (Table 5e). Invasive species removal experiments, perhaps in conjunction with deer exclosure experiments, could distinguish the effects of direct competition, deer‐mediated competition (“apparent competition” sensu Holt, 1977), or mere correlation between native species declines and invasions of introduced plant species (Didham et al., 2005).

The metabolomic novelty of the invasive species considered here was measured relative to the plant community at a single site, the SERC 16‐ha forest plot in Maryland. Hence, our tests of the predictions of the novel weapons and enemy release hypotheses would be improved by expanding this analysis of the chemical similarities of invasive and native plants to multiple plant communities in a broader geographic region. Invasive species could be chemically distinct in their introduced ranges simply because of extrinsic differences between the plant communities in which they are native or exotic, or alternatively, because the invasive species exhibit intrinsic characteristics, unique chemistry, that make them exceptionally well‐defended against natural enemies regardless of community context (Colautti et al., 2014). To discriminate between these alternative hypotheses, one would need to compare the metabolomic similarity of invasive plants to the flora within their native, primarily Asian, distributions, and plant communities to which they are introduced and in which they are invasive.

The Japanese beetle shares its native range with many of the invasive species considered here and may have coevolved with them. The relative preference for particular plant species differed between the Japanese beetle and the native woolly bear caterpillar, but the two insect herbivores exhibited remarkably similar relationships between diet preference and chemical distinctiveness of both native and invasive plant species in Maryland (Figure 5a,b). This result lends support to the hypothesis that many invasive plant species exhibit intrinsic chemical properties that make them chemically distinct and well‐defended in both their native range and in communities to which they are introduced.

### 4.2 Future directions

Our study used LC‐MS methods appropriate for nonvolatile compounds. Volatile organic compounds (VOCs) are known to influence host plant choice through olfactory signaling and defense (Raguso et al., 2015). Volatile compounds are typically analyzed using gas chromatography–mass spectrometry (GC‐MS), but such data can now be integrated into the molecular networking metabolomics methods used here.

We focused diet assays on only two generalist insect herbivores and browse surveys only on white‐tailed deer. White‐tailed deer are likely the dominant herbivore affecting plant performance and variation in demography in eastern North America (Averill et al., 2018). However, insect herbivores and microbial pathogens also exert pressure on plant fitness, and their influence on plant survival and recruitment likely supersedes that of mammalian herbivores in warmer and wetter climates (Mangan et al., 2010; Schemske, Mittelbach, Cornell, Sobel, & Roy, 2009). Future studies should expand on the present study to examine the contribution to biological invasions of both volatile and nonvolatile compounds and their effects on diverse plant enemies that include generalist and clade‐specialist herbivores, as well as microbial pathogens.

## CONFLICT OF INTEREST

The authors declare no conflicts of interest.

## AUTHOR CONTRIBUTIONS

**Brian E. Sedio:** Conceptualization (lead); data curation (lead); formal analysis (lead); funding acquisition (lead); investigation (equal); methodology (lead); visualization (lead); writing – original draft (lead); writing – review and editing (equal). **John L. Devaney:** Conceptualization (supporting); data curation (supporting); formal analysis (supporting); investigation (equal); methodology (supporting); writing – review and editing (equal). **Jamie Pullen:** Data curation (supporting); investigation (equal); methodology (supporting). **Geoffrey G. Parker:** Conceptualization (supporting); data curation (supporting); funding acquisition (supporting); investigation (equal); methodology (supporting); writing – review and editing (equal). **S. Joseph Wright:** Conceptualization (supporting); funding acquisition (supporting); methodology (supporting); writing – review and editing (equal). **John D. Parker:** Conceptualization (supporting); data curation (supporting); funding acquisition (supporting); investigation (equal); methodology (supporting); supervision (supporting); writing – review and editing (equal).

## Data Availability

The SERC ForestGEO 16‐ha plot data are publicly available through the ForestGEO data portal: http://ctfs.si.edu/datarequest/ The USDA FIA invasive species plot census data are publicly available through the FIA data portal: https://apps.fs.usda.gov/fia/datamart/datamart.html Mass spectra and molecular networks are publicly hosted on the GNPS database at http://gnps.ucsd.edu/ProteoSAFe/status.jsp?task=d1f7f083fa554f2c9608f238c1ccda0e and https://gnps.ucsd.edu/ProteoSAFe/status.jsp?task=f029d6c3192341b990879ffe120881b4. Data reported in Tables 1 and 2 are archived on Dryad at https://doi.org/10.5061/dryad.w3r2280ng.
